# Impact of interventions to prevent anxiety and depression in people with inflammatory rheumatological conditions: a systematic review

**DOI:** 10.1093/rap/rkag059

**Published:** 2026-05-29

**Authors:** Lauren Gray, Matilda Bjorklund, Samantha L Hider, Tom Kingstone, Carolyn A Chew-Graham, Nadia Corp

**Affiliations:** School of Medicine, Keele University, Keele, UK; Research and Innovation, Midlands Partnership University NHS Foundation Trust, Staffordshire, UK; School of Medicine, Keele University, Keele, UK; School of Medicine, Keele University, Keele, UK; Research and Innovation, Midlands Partnership University NHS Foundation Trust, Staffordshire, UK; School of Medicine, Keele University, Keele, UK; Research and Innovation, Midlands Partnership University NHS Foundation Trust, Staffordshire, UK; School of Medicine, Keele University, Keele, UK; Research and Innovation, Midlands Partnership University NHS Foundation Trust, Staffordshire, UK; School of Medicine, Keele University, Keele, UK

**Keywords:** systematic review, mental health, anxiety, depression, inflammatory arthritis, prevention

## Abstract

**Objectives:**

Anxiety and depression are associated with poorer outcomes for people with inflammatory rheumatological conditions (IRCs). A systematic review of psychosocial interventions to prevent anxiety and/or depression in people with long-term conditions was conducted. This analysis aimed to explore the effectiveness of such interventions in IRCs and the subsequent impact on condition and quality of life.

**Methods:**

A systematic review examining psychosocial interventions to prevent anxiety and/or depression in people with IRCs. Studies reporting a psychosocial intervention for IRCs and no clinical diagnosis of anxiety or depression at baseline were included. Seven databases (MEDLINE, EMBASE, AMED, CINAHL Plus, PsycINFO, Cochrane Central, Web of Science Core Collection) were searched.

**Results:**

The search identified 24 001 unique papers, with 46 being included in the main review. Twelve of these focused on IRCs and are reported in this paper: 10 Randomised Controlled Trials (RCT) and 2 non-RCTs with 830 participants. Three categories of interventions were identified: psychological therapies (*n* = 5), exercise (*n* = 3) and educational programmes (*n* = 4). Eight studies reported a positive effect on anxiety and/or depression prevention. Three of these studies also reported an improvement in fatigue. Only one study reported a positive impact on pain outcomes. Only one study showed improvement in disease activity. Studies predominantly focused on depression outcomes; anxiety was rarely the primary focus. Studies generally scored very low/low certainty of evidence.

**Conclusions:**

Offering psychological therapies may help prevent anxiety and/or depression in people with IRCs and may also lead to improvements in fatigue. Given the impact of mood and fatigue on outcomes, further high-quality studies are needed.

Key messages

- Psychological therapies, when offered early, may help prevent anxiety and depression in people with IRCs.
- Effective interventions to prevent anxiety and/or depression may also help improve fatigue and physical functioning.
- More research is needed into anxiety and depression prevention in IRCs, particularly anxiety.

## Introduction

Adults with inflammatory rheumatological conditions (IRCs), including RA, AS, PsA and PMR, are at an increased risk of anxiety and depression [1, 2]. The prevalence of depression is estimated to be between 16.8% and 38.8% for RA [1], 11% to 64% for AS [3] and 9% to 22% for PsA [4]; the prevalence of anxiety is less well characterized [4–7]. This increased risk adversely impacts physical health outcomes [8] and quality of life [8, 9] and is associated with increased health care costs [10] and decreased treatment concordance [11, 12].

Living with an IRC can be life changing. A diagnosis of a long-term condition (LTC) can cause shock, uncertainty and re-evaluation of the future [13]. People often require lifestyle changes and adoption of new self-management behaviours to reduce the condition’s impact [14]. Psychosocial interventions may help support people living with a LTC [14].

Given the negative impact of anxiety and depression on people with LTCs, anxiety/depression prevention strategies are likely important. This review considers primary prevention (designed to decrease the number of new symptoms) and secondary prevention strategies (designed to lower the rate of existing symptoms) [15].

Prevention interventions may include pharmacological or psychosocial interventions, such as psychological approaches, health education, social support or physical exercise.

There is systematic review evidence for effectiveness of depression-prevention strategies for diabetes [16] whereby incorporating a psychological component with an educational approach was beneficial to well-being and prevented depression [16]. There is also pharmacological evidence to prevent depression in coronary syndromes and stroke [17]. There is a systematic review of interventions to reduce depression symptoms in people with RA (although not focused on prevention specifically) [18] but little for other IRCs.

Currently, NICE provide recommendations for the recognition and management of depression, but not anxiety, in adults with chronic physical health problems [19], which are reflected in NICE disease-specific treatment [20, 21] guidelines. However, these focus on the treatment of already diagnosed depression and anxiety disorders, rather than prevention.

To explore the evidence of interventions for prevention of anxiety and depression, we undertook a systematic review across LTCs. This paper synthesizes a subset of data related to inflammatory conditions only.

## Methods

This systematic review was conducted and is reported following the Cochrane Handbook for Systematic Reviews [22] and ‘Preferred Reporting Items for Systematic Reviews and Meta-Analyses 2020’ (PRISMA 2020) guidelines [23]. The full protocol was registered with the International prospective register of systematic reviews (PROSPERO: CRD42022333954). Screening and data extraction were completed as part of the whole review, with then 12 studies focused on IRCs being explored separately within this paper.

### Search strategies

Comprehensive search strategies were implemented in seven electronic databases: MEDLINE (Ovid), EMBASE (Ovid), AMED (Ovid), CINAHL Plus (EBSCO), PsycINFO (EBSCO), Cochrane Central and Web of Science Core Collection from inception to October 2024 (see Table S1, available at *Rheumatology Advances in Practice* online, for full searches).

Results were imported into RefWorks (reference management software, ProQuest) and duplicates removed. Unique records were imported into Rayyan to manage the screening process.

### Study eligibility

Any controlled study that reported a psychosocial intervention involving adults (≥18 years) with an IRC (RA, AS, PsA, PMR, SLE) and had no clinical diagnosis of anxiety and/or depression at baseline but were assessed for depression and/or anxiety as an outcome were included (Table 1).

**Table 1:** Eligibility criteria following population intervention comparison outcome (PICO) format.

Population or participants and conditions of interest	Aged ≥ 18 years Diagnosed with any IRC: RA Axial spondylarthritis PsA PMR SLE
Interventions	Any psychosocial intervention
Comparisons or control group	A comparator/control group of usual care, placebo or waiting list control
Outcomes of interest	A reduction of depressive or anxiety symptoms measured by a standardized validated tool OR Onset of a depression or anxiety (clinical diagnosis)
Study designs	Any controlled study designs
Exclusion criteria	Studies including only people who have a reported clinical diagnosis of depression and/or anxiety at baseline Studies with people who are not community dwelling/living independently Studies that evaluate pharmacological interventions Non-English language articles for which an interpreter could not be found within the timeframe of the review

IRC: inflammatory rheumatological condition.

### Screening process

Titles were screened by a single reviewer and those clearly irrelevant excluded. Abstracts were independently screened by two reviewers, against eligibility criteria: inter-rater reliability score was high (kappa coefficient ≥0.80). Full text screening was completed by one reviewer (L.G.), with a second reviewer (M.B.) independently screening a 25% sample. Disagreements were minimal, and were resolved through discussion, or by referral to a third reviewer (N.C.).

### Data extraction and risk of bias

A customized and piloted data extraction form was developed in Excel and was used to record all data for analysis. Data extracted included: country, population characteristics, intervention characteristics informed by the TIDieR checklist [24], outcome measures at baseline and all follow-up time points, intervention adherence and fidelity, and summary effect estimates. Data were extracted by one reviewer, with 50% cross-checked by a second reviewer. Risk of bias was assessed using Cochrane Risk of Bias v1 for RCTs [25] and ROBINS-I for other controlled studies [26] (L.G.) and cross-checked by M.B.

### Data analysis and evidence synthesis

Due to significant heterogeneity in intervention types, populations and outcomes among studies, a meta-analysis was not feasible, therefore, a narrative synthesis was conducted [27]. The synthesis focused on identifying components and characteristics of psychosocial interventions that demonstrated a positive effect on anxiety and/or depression. The synthesis began with a description of study characteristics and main results presented in summary tables, followed by the extraction and tabulation of intervention components to facilitate comparison.

The overall strength of evidence for intervention effectiveness was assessed using a GRADE-informed approach [28] (see Table S2, available at *Rheumatology Advances in Practice* online, for criteria used). The overall certainty of evidence for each outcome was then rated independently by L.G. and M.B. and compared. Any discrepancies were discussed and resolved.

## Results

### Study characteristics

**Figure 1:**
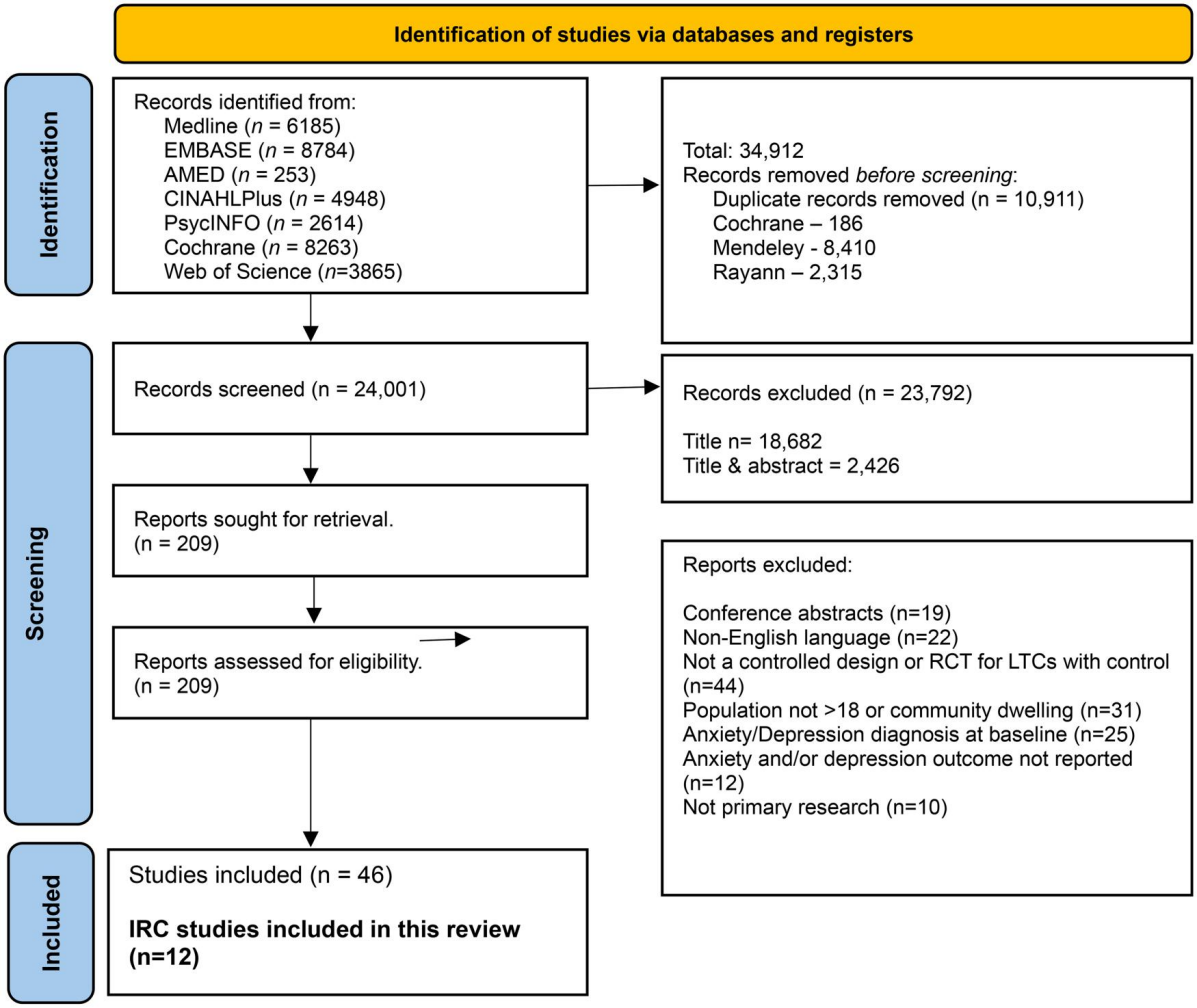
PRISMA flowchart. Source: Page MJ, et al. BMJ 2021;372:n71. doi: 10.1136/bmj.n71. For more information, visit: http://www.prisma-statement.org/

The search yielded 24 001 unique records, of which 209 were included for full-text screening. Of these 46 met inclusion criteria and 163 were excluded for reasons given in Fig. 1. Twelve studies considered IRCs and are the focus for this paper.

Studies included were published between 1997 and 2024, and from nine different countries. The majority were RCTs (*n* = 10). Study participants were individuals with RA (*n* = 4), SLE (*n* = 4), AS (*n* = 3) or mixed inflammatory conditions (*n* = 1). Across all studies the sample size was 830 (range 28–142) with mean ages between 29.93 and 59.43 years. The mean percentage of females was 60.5% (range 18–100%). Demographics are presented in Table 2.

**Table 2:** Study characteristics.

Author year (country) Study design	Intervention	Participants (N, age (SD), % female)	Intervention duration	Follow-up	Baseline measures of depression and anxiety symptom severity	Treatment effects Depression	Treatment effects Anxiety	Treatment effects Impact on condition	Treatment effects Quality of life	Risk of bias
Mixed										
Baglan-Yentur 2024 (Turkey) Non-RCT	Remote exercise programme	28 39.75 (0.7) 85.7%	8 weeks	Post-intervention	HADS-D: 8.81 ± 5.29 HADS-A: 11.87 ± 5.28	HADS-D Mann–Whitney IG Δ −1.25 ± 6.800 (−19-12), Control Δ −0.33 ± 4.00(−9-8), *P* = 0.423	HADS-A Mann–Whitney: IG Δ −2.81 ± 4.33 (−14-7), Control Δ −0.66 ± 4.97(−10-6), *P* = 0.223	FSS: IG Δ −3.43 ± 3.42 (−11-4), Control Δ 0.41 ± 11.34 (−13-26), *P* = 0.725 Clinical disease activity for RA: No *P*-value reported BASDAI: no *P*-value reported BASFI: IG Δ −0.8 ± 0.4 (−1.2 - −0.4), Δ 0.6 ± 1.6 (−0.5-1.7), *P* > 0.05 SLE global assessment: no *P*-value reported	QoL (HAQ): IG Δ −0.32 ± 0.78 (−2-0.7), Control Δ 0.83 ± 0.47 (−0.50-1.20), *P* = 0.272	**Moderate**
RA										
Barlow 1997 (UK) RCT	Patient Education Leaflet	142 59.43 (11) 81.48%	N/A	3 weeks	HADS-D: 6.56 ± 4.23 HADS-A: 7.85 ± 5.09	HADS-D ANOVA (F = 3.64, *P* = 0.059)	HADS-A ANOVA (*P* = 0.960)	**Pain (VAS): ANOVA F = 6.45, *P* = 0.013**	Not assessed	High
								Fatigue (VAS): ANOVA *P* = 0.226		
Dalili 2019 (Iran) RCT	MBCT	28 NR NR	8 weeks	Post-intervention	DASS-21 Depression: 15.71 ± 2.52 DASS-21 Anxiety: 12.78 ± 2.91	**DASS-21 Depression MANOVA (F = 60.56, *P* < 0.001)**	**DASS-21 Anxiety MANOVA (F = 48.85, *P* < 0.001)**	Not assessed	Not assessed	High
Evers 2002 (Netherlands) RCT	Tailor-made CBT	64 53.7 (11.39) 73.5%	24 weeks	6 and 12 months	BDI-II: 12.79 ± 6.46 IRGL Anxiety: 22.23 ± 5.26	**BDI-II univariate test (_F2,5 5_ =5.34, *P* < 0.01)**	**IRGL anxiety univariate test (F_2,5 5_ =0.67, *P* = 0.52) 6 months (t = 3.46, *P* < 0.01) 12 months (t = 1.63, *P* = 0.11)**	**Physical functioning (all) functional disability, pain, fatigue: multivariate analyses F_(6,51)_ =3.16, *P* < 0.05.**	Not assessed	High
								Functional disability (IRGL + mobility/self-care scales): univariate analyses F_(2,55)_=1.82, *P* = 0.17		
								Pain (IRGL Pain Scale): univariate analyses F_(2,55)_ =0.27, *P* = 0.77		
								**Fatigue (Fatigue scale Checklist Individual Strength): univariate analyses F_(2,55)_=4.17, *P* < 0.05**		
								Disease Activity (erythrocyte sedimentation rate and clinical joint score): general linear model repeated measures (F_(2,55)_=2.03, *P* = 0.14)		
Sharpe 2003 (UK) RCT	CBT	53 55.06 (14.07) 71%	8 weeks	Post-intervention, 6 months and 18 months	HADS-D: 4.87 ± 4.01 HADS-A: 8.65 ± 5.6	**HADS-D MANCOVA. post-test (F_(1,41)_=6.041, *P* = 0.018). 6 months (F_(2,41)_=3.915, *P* = 0.05). 18 months (F_(2,51)_=5.357, *P* = 0.012).**	**HADS-A MANCOVA. post-test (F_(1,41)_=3.2547, *P* = 0.086). 6 months (F_(2,41)_=2.522, *P* = 0.078). 18 months (F_(2,51)_=4.378, *P* = 0.02)**	**Joint inflammation : F_(2,51)_=5.181, *P* = 0.01 over three assessments, but no difference between the groups at follow-up F_(2,51)_=0.385, *P* > 0.4.** **Pain (Likert scale):** F_(2,51)_=2.577, *P* = 0.115 erythrocyte sedimentation rate: F_(2,51)_=1.274, *P* = 0.264 CRP: F_(2,51)_=0.90, *P* ≥ 0.40, but not maintained at follow-up F_(2,38)_=2.801, *P* = 0.067 Pain (Likert scale): F_(2,51)_=2.577, *P* = 0.115	Not Assessed	Unclear
AS										
Durmus 2009 (Turkey) Non-RCT	Home-based exercise programme	43 39.83 (8.23) 19%	12 weeks	Post-intervention	BDI-II: 9.24 ± 3.17	**BDI-II independent samples t-test (*P* = 0.001).**	Not assessed	**Functional capacity (BASFI): *P* = 0.001** **Disease activity (BASDAI): *P* = 0.001**	**Short Form 36:** Overall *P* < 0.05	**Moderate**
								**Fatigue (MAF): *P* = 0.003**		
								**Pain (SF-36): *P* = 0.001**		
Kaya 2016 (Turkey) RCT	Peer-led group education	80 42 (9.2) 18%	4 weeks	Post-intervention and 6 months	BDI-II: 9 (1–34)	BDI-II Friedman test. IG; *P* = 0.310, Control; *P* = 0.075	Not assessed	Disease activity (BASDAI): data not shown Physical functioning (BASFI): data not shown	SF-36: All domains not significant ASQoL: not significant	**High**
Song 2021 (China) RCT	Educational Intervention via WeChat	118 29.93 (8.23) 21.2%	12 weeks	Post-intervention	BDI-II: 5.00 (10.00)	**BDI-II Mann–Whitney U test (Z = 1.980, *P*= 0.048).**	Not assessed	Functional index (BASFI): −1.764, *P* = 0.078 **Bodily pain (SF-36): t = 3.340, *P* = 0.001** Pain (VAS): overall −0.583, *P* = 0.561 Back pain: 0.658, *P* = 0.512 Nocturnal pain: −0.086, *P* = 0.932 Morning stiffness: −0.672, p0.503	**SF-36 Role on limitations physical: (Z =** −**2.429, *P* = 0.015)** **SF-36 General health: (t = 2.565, *P* = 0.012)** **SF-36 Social functioning: (t = 2.917, *P* = 0.004)** **SF-36 Role on limitations mental: (t = 2.797, *P* = 0.006)** **SF-36 Mental health: (t = 2.534, *P* = 0.013)** SF-36 Vitality: (t = 1.600, *P* = 0.112) SF-36 Physical functioning: (t=0.240, *P* = 0.217) SF-36 Mental health: (t = 2.534, *P* = 0.013)	**Unclear**
SLE										
Conceição 2019 (Brazil) RCT	Brief Group Psychoanalytic Psychotherapy	80 42.4 (11.7) 100%	20 weeks	Post-intervention	HADS-D: 8.0 (0.0–14.0) HADS-A: 9.0 (0.0–20.0)	**HADS-D Mann–Whitney inter-group; baseline *P* = 0.264, 20 Week *P* = 0.022)**	**HADS-A Mann–Whitney inter-group; baseline *P* =** −**0.340, 20 weeks *P* = 0.019**	SLEDAI: Mann–Whitney (inter-group; baseline *P* = 0.347, 20 weeks *P* = 0.207) **SLE-SSC: Mann–Whitney (inter-group; baseline *P* = 1.01, 20 weeks *P* < 0.001).**	**SLE-QOL: Mann–Whitney (*P* = 0.041)**	**High**
Gavilán-Carrera 2022 (Spain) Non-RCT	Aerobic Exercise Programme	58 44 (13.9) 100%	12 weeks	Post-intervention	BDI-II: 9.6 ± 7.7	BDI-II quantile regression (mean difference = −1.78, 95% CI = −6.74 to 3.19, d = 0.21, *P* = 0.475)	Not assessed	**Fatigue (MAF): general fatigue (**−**2.86;** −**5.70 to** −**0.01, *P* = 0.049, d = 0.57)** **MAF physical fatigue: (**−**4.33;** −**7.02 to** −**1.65; *P* = 0.002, d = 0.92)** MAF motivation: (−1.29; −2.60 to 0.03; *P* = 0.055, d = 0.60) MAF reduced activity: (*P* = 0.839, d = 0.06)	SF-36 Physical component: (2.34; −3.83 to 8.52, *P* = 0.448, d = 0.14) SF-36 Mental component (4.39; −3.00 to 11.78, *P* = 0.237, d = 0.27)	**High**
Sakr 2022 (Egypt) RCT	Psychoeducational Therapy	80 32.3 (8.7) 96.25%	10 sessions	3 months	Symptom Checklist 90 Depression: 0.88 ± 0.47 Symptom Checklist 90 Anxiety: 0.31 ± 0.36	**Symptom Checklist 90 depression independent samples t-test (*P* = 0.002).** But also significant difference between groups at baseline (*P* = 0.04)	**Symptom Checklist 90 anxiety independent samples t-test (*P* = 0.05)**	SLE disease activity: independent samples t-test (*P* = 0.80). Physical functioning (SF-36): independent samples t-test (*P* = 0.25) Fatigue (SF-36): independent samples t-test (*P* = 0.09) Pain (SF-36): independent samples t-test (*P* = 0.08)	**SF-36 Role of limitations due to mental (*P* < 0.001)** **SF-36 Emotional well-being (*P* < 0.001)** **SF-36 Mental Component Summary (*P* = 0.001)** **SF-36 Physical Component Summary (*P* = 0.03)** SF-36 Role of limitations due to physical (*P* = 0.09)	**Unclear**
									SF-36 Social Functioning (*P* = 0.1) SF-36 General Health (*P* = 0.63)	
Sohng 2003 (Korea) Non-RCT	SLE Self-Management Course	56 32.6 (11.2) NR	6 weeks	Post-intervention	BDI-II: 12.8 ± 9.1	**Wilcoxon signed-rank sum test BDI-II (mean change (95% CI) = 4.3 (0.14 to 8.48), *P* = 0.025)**	Not assessed	**Wilcoxon signed-rank sum test mean change** **Fatigue (multidimensional assessment of fatigue): 6.7 (**−**0.46, 9.24), *P* = 0.049**	Not assessed	Critical
								Pain (VAS): 0.1(-0.45, 0.31), *P* = 0.469		
								Disease activity: C3 - 6.4(9.58, 20.18), *P* = 0.973, C4 −2.8 (−3.35, 9.49), *P* = 0.696, anti-ds −58.5 (38.71, 142.13), *P* = 0.273		

Bold text indicates statistically significant results.

BAI = Beck Anxiety Inventory

BASDAI = Bath Ankylosing Spondylitis Disease Activity Index

BASFAI = Bath Ankylosing Spondylitis Functional Index

BDI = Beck Depression Inventory

CBT= Cognitive Behavioural Therapy

CRP = C-Reactive Protein

DASS-21 = Depression, Anxiety and Stress Scale

FSS = Fatigue Severity Scale

HADS = Hospital Anxiety and Depression Scale

HAQ = Health Assessment Questionnaire  IRGL = Impact of Rheumatic diseases on General health and Lifestyle

MAF = Multidimensional Assessment of Fatigue

MBCT = Mindfulness Based Cognitive Therapy

N = number

QoL = Quality of Life

SD = standard deviation

SF-36 = Short-Form 36

SLEDAI = Systemic Lupus Erythematosus Disease Activity Index

SLE-SSC = Systemic Lupus Erythematosus - Systemic Sclerosis/Scleroderma

VAS = Visual Analogue Scale

### Risk of bias

**Figure 2:**
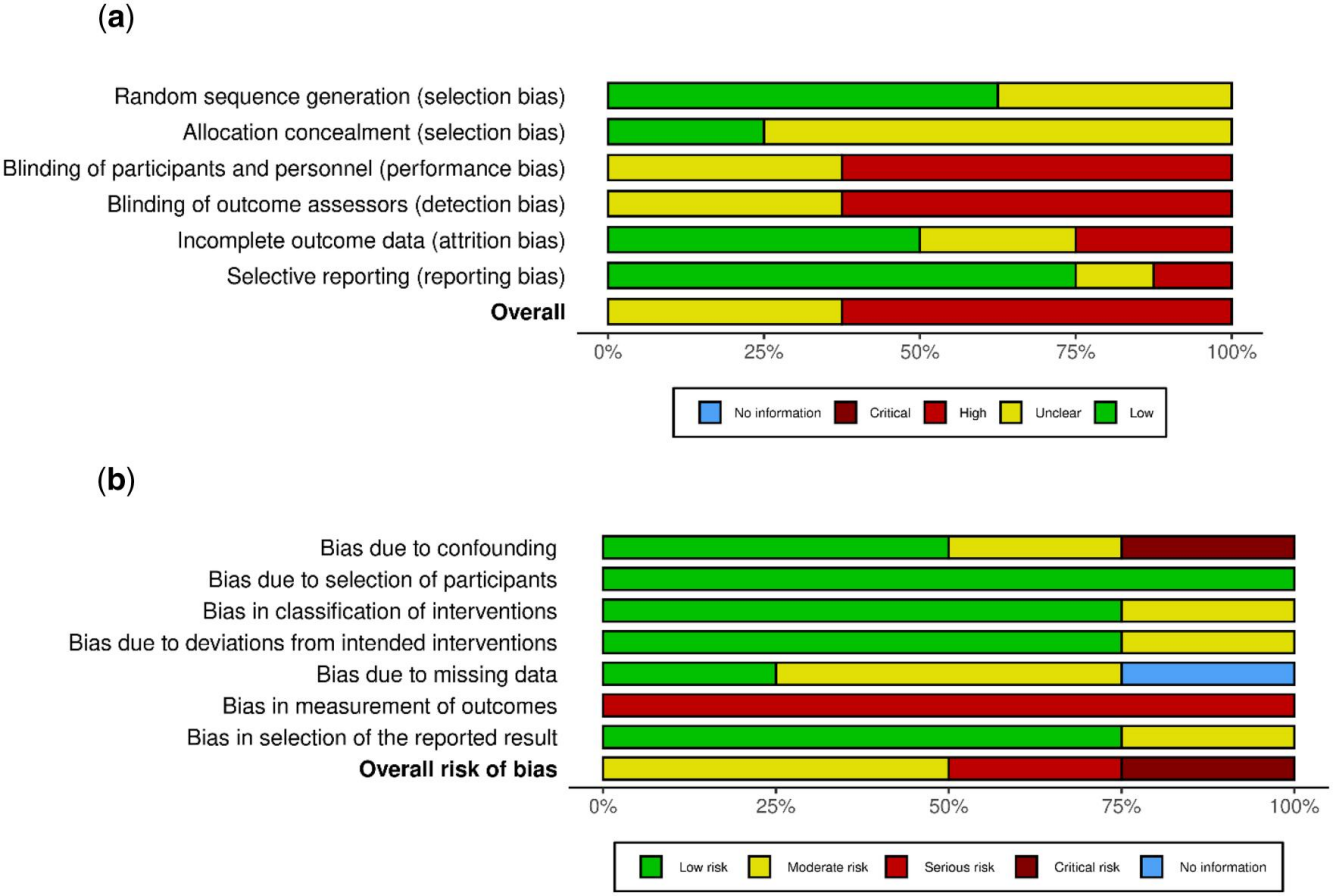
Aggregated risk of bias for Cochrane risk of bias (a) and ROBINS-1 (b)

Overall risk of bias across RCTs was ‘unclear’ (37.5%) to ‘high’ (62.5%) (Table 2, Fig. 2). This was mainly due to lack of blinding of participants, study personnel or outcome assessors (5/8) with the others being unclear (Fig 2 and Fig. S1, available at *Rheumatology Advances in Practice* online). Overall, risk of bias on non-RCT studies ranged from ‘moderate’ to ‘critical’ (Fig. 2). This was due to differences in baseline characteristics (2/4), missing data (3/4) and insufficient blinding of outcome measures (4/4) (Fig. 2 and Fig. S2, available at *Rheumatology Advances in Practice* online).

### Preventative interventions on anxiety and depression in IRCs

Interventions investigated included cognitive behavioural therapy (CBT) [29, 30], mindfulness-based cognitive therapy (MBCT) [31], group psychoanalytic therapy [32], exercise programmes [33–35] and educational based programmes, including patient leaflet [36], peer group led education [37], self-management interventions [38, 39] and psychoeducation [39].

#### Depression prevention

Eight of twelve studies reported a significant intervention effect. Effective interventions included CBT [29, 30], MBCT [31] Group Psychoanalytic Psychotherapy [32], a home-based exercise programme [34,] psychoeducation [40] and self-management and educational courses [37–39].

CBT interventions [29, 30] and the MBCT intervention [31] found positive effect for depression prevention in RA; there was no evidence for CBT or MBCT in other IRCs. Group psychoanalytic therapy found a positive effect on depression prevention in people with SLE [32].

One of three exercise programmes included had a positive effect on depression prevention [34]. This was a home-based exercise programme including muscle strengthening and posture exercises, and relaxation in people with AS. However, two exercise interventions using a live video session [33] or an in person aerobic exercise session [35] did not impact depression prevention.

Of the educational interventions, two self-management programmes, one using a six-week face-to-face course for people with SLE [38] and one using online messaging application to manage AS [39] reported significant effects on depression prevention [38, 39]. Psychoeducation also had positive effects on depression prevention in people with SLE [40]. However, a peer group education programme [37] did not show an effect for depression prevention for AS, and neither did postal educational materials, suggesting that type of education and mode of delivery may be important.

The certainty of evidence for depression prevention in people with IRCs is low (Table 3). While there is a sufficient number of studies showing positive effects (*n* = 8 of 12), these do not have adequate sample sizes, do not report or report low effect sizes, and all have high or moderate/unclear risk of bias.

**Table 3:** Certainty of evidence.

Certainty of evidence informed by GRADE								
	References	No. of studies	Total no. of participants	Concerns regarding			Factors that may increase quality	
				Risk of bias	Consistency	Precision	Magnitude	Overall certainty of evidence
Depression	Baglan-Yetur 2024, Barlow 1997, Conceição 2019, Dalili 2019, Durmus 2009, Evers 2002, Gavilán-Carrera 2022, Kaya 2016, Sakr 2022, Sharpe 2003, Sohng 2003, Song 2021	12	830	**Concern:** 8/12 were RCTs, 4/12 were non-RCTs. 5/8 RCT studies have high risk of bias, and 3/8 RCTs were unclear. 1 Non-RCT critical risk of bias, one with serious and two with moderate.	No concern: 8/12 studies showed significant improvement.	**Concern:** 10/12 studies have <50 per arm.	**Concern:** 12/12 studies do not report or report low effect size.	++ Low
Anxiety	Baglan-Yetur 2024, Barlow 1997, Conceição 2019, Dalili 2019, Evers 2002, Sakr 2022, Sharpe 2003	7	475	**Concern:** 6/7 were RCTs, 1/7 were non-RCTs. 4/6 RCTs have high risk of bias, 2/6 have unclear. 1 Non-RCT had moderate risk of bias.	No Concern: 5/7 studies showed significant improvement.	**Concern:** 6/7 studies have <50 per arm.	**Concern:** 9/9 studies do not report or report low effect size.	++ Low

Bold text indicates concern. RCT = Randomized Controlled Trial.

#### Anxiety prevention

Seven out of the 12 studies reported anxiety as an outcome. These included CBT interventions [29, 30], MBCT [31 ]and group psychoanalytic therapy [32], an exercise programme [33], and two educational interventions [36, 40].

Both CBT interventions [29, 30] and the MBCT intervention [31] found positive effect for anxiety prevention in people with RA, with group psychoanalytical therapy having positive effects in people with SLE [32]. The single exercise intervention, using physiotherapist videocall-delivered exercise [33], did not prevent anxiety.

The only exercise intervention that reported anxiety in IRCs was a remote physiotherapist exercise programme using videocall [33]. However, this did not find any significant effects on anxiety prevention.

Psychoeducation [40] found positive effects on anxiety prevention in people with SLE. But, again, distributing educational materials by post was ineffective for anxiety [36].

There is low certainty of evidence for anxiety prevention in people with IRCs (Table 3). Of the seven studies that reported anxiety, six were RCTs and one was a non-RCT which all showed moderate, high or unclear risk of bias. Most of the studies showed significant improvement (*n* = 5/7), but did not have sufficient sample size or did not report or reported low effect size.

### Intervention components and impact on depression or anxiety prevention

Twelve distinct psychosocial intervention components were identified (see Table 4 and Table S3, available at *Rheumatology Advances in Practice* online). Six incorporated education or a mindfulness and/or relaxation component, five interventions (42%) involved cognitive restructuring, and four (33%) focused on communication and relationship development. Other interventions included problem-solving, attention training, exercise and peer support (*n* = 3 for each). Two interventions (16.7%) featured reflective practice, or homework, and one intervention (8.3%) included goal setting or taught an element of acceptance. Overall, three interventions (25%) used one component, three interventions (25%) used two components and six interventions (50%) used more than two components.

**Table 4:** Intervention components.

	Problem-solving	Cognitive restructuring	Attention training	Communication and relationship development	Mindfulness and relaxation	Exercises	Reflection	Peer support	Education and information	Homework	Acceptance	Goal setting
Baglan-Yentur (2024)					**✓**	**✓**						
Barlow (1997)									**✓**			
Conceição (2019)^a^		**✓**						**✓**	**✓**			
Dalili (2019)^a^		**✓**	**✓**		**✓**		**✓**			**✓**	**✓**	
Durmus (2009)^b^						**✓**						
Evers (2002)^a^	**✓**	**✓**	**✓**	**✓**	**✓**							
Gavilán-Carrera (2022)					**✓**	**✓**						
Kaya (2016)								**✓**	**✓**			
Sakr (2022)^a^	**✓**	**✓**		**✓**	**✓**				**✓**			**✓**
Sharpe (2003)^a^	**✓**	**✓**	**✓**	**✓**	**✓**							
Sohng (2003)^b^				**✓**					**✓**			
Song (2021)^b^							**✓**	**✓**	**✓**	**✓**		

Highlighted in shaded rows:

a Significant effects for depression and anxiety.

b Significant effects for depression only.

#### Depression prevention

Effective components included cognitive restructuring (*n* = 5), education and information (*n* = 4) and communication and relationship development (*n* = 4). Those studies which did not show effect used mindfulness/relaxation (*n* = 2), exercise (*n* = 2), education and information (*n* = 2) and peer support (*n* = 1) suggesting exercise and mindfulness components may not be the most effective way to prevent depression although numbers are small. Also, mixed effects were seen for education and information, with significant effects in four studies [31, 37, 38, 39] but no significant effects in two others [35, 36], suggesting that differences in the delivery and type of information provided may influence intervention effectiveness.

The interventions that reported significant effects for depression prevention used a median (interquartile range [IQR]) of 5 (3) components compared with ineffective interventions which used a median of two components (IQR = 0.5), Mann–Whitney U test (*P* = 0.049).

#### Anxiety prevention

In the studies that found positive effects on anxiety outcomes (*n* = 5), all used cognitive restructuring and four also used mindfulness or relaxation (*n* = 4). Those studies that did not have significant effects on anxiety (*n* = 2) reported a mix of mindfulness (*n* = 1), exercise (*n* = 1) and education/information (*n* = 1).

The interventions that reported significant effects for anxiety prevention used a median of five components (IQR = 2) whereas those ineffective for anxiety prevention used a median of 1.5 components, although this was not statistically significant (Mann–Whitney *P* = 0.076).

### Effect of interventions to prevent anxiety/depression on other disease outcomes

Seven studies reported physical outcomes in patients with SLE (*n* = 3), RA (*n* = 2) and AS (*n* = 2) (Table 2).

Pain was reported in six studies (Table 2). Only one non-RCT found that effective interventions for anxiety and/or depression also improved pain reporting [33], the majority (*n* = 5) reported no effect [28, 29, 37–39].

Four studies reported fatigue (Table 2) with three showing improvements in fatigue [28, 33, 37], although one study [39] did not find any improvement in fatigue.

Physical functioning was reported in four studies (Table 2). The evidence for physical functioning was mixed: two studies found that the intervention led to improvements in physical functioning [28, 33] while two reported no improvement [38, 39].

Disease activity was reported by six studies (Table 2), with five studies showing no effects [28, 29, 31, 37, 39] and one study found positive effects [33].

## Discussion

This systematic review evaluated psychosocial interventions to prevent anxiety and/or depression for people with IRCs and assessed the impact on the index condition. Findings suggest there is some evidence for depression prevention; however, the certainty of evidence is low and there is considerable heterogeneity in the published data.

The findings suggested that multicomponent interventions with more components were more effective than multicomponent interventions with fewer components for both anxiety and depression prevention. Different combinations of components may be more effective than others. Effective components included education and information and communication and relationship development for depression prevention, mindfulness for anxiety prevention and cognitive restructuring for both. Mixed results were seen for education interventions with positive findings for self-management programmes but no effect with posted information leaflets suggesting mechanism of delivery may be important.

This review also suggests that interventions that aim to prevent anxiety and/or depression can have a positive effect on fatigue experienced by patients which may lead to improvements in quality of life. Among the studies that were effective for depression prevention, only one study showed improvement in pain and the impacts on physical functioning were mixed. Few studies used patient reported disease activity measures.

This systematic review has provided a global perspective on psychosocial interventions for people with IRCs. A comprehensive, systematic search strategy was developed with an experienced information specialist and two reviewers performed screening and data extraction with high inter-rater reliability scores enabling confidence in the findings with assessment of study quality using validated tools. The studies in this systematic review included a variety of intervention types, populations and outcomes; hence, a meta-analysis was inappropriate, and a narrative synthesis used to synthesize results.

There were some methodological challenges especially defining the concept of ‘prevention’ which, as previously discussed, lacks clear definition and conceptualization in the literature. Often, randomized controlled trials aiming at examining a preventative intervention do so by measuring reduction of symptoms which makes prevention difficult to distinguish from treatment studies. To ensure consistency, studies that included people with an existing diagnosis of anxiety and/or depression were excluded. The findings highlight the poor study quality and risk of bias.

Previous systematic reviews have shown evidence for depression prevention in older adults or in people with macular degeneration [17] that have focused on other LTC but have found similar types of psychosocial and interventions [40], although this focused on older adults only. A review of depression prevention [17] found very low certainty of evidence for prevention interventions although this only included one psychosocial intervention.

The data support the need for complex interventions to address depression prevention, since a greater number of components was associated with effective interventions, which align with the current research agenda that complex interventions are needed to tackle managing people with multiple LTCs [41, 42]. Whether these components were effective independently or the interactions between components led to success, it is not clear. But these findings have provided insights on which combinations may work together.

This review also examined the impact of the effective interventions on disease-specific outcomes finding that effective interventions improved fatigue reporting. This is consistent with the current evidence that there is a strong relationship between mood and fatigue, with low mood frequently associated with increased levels of fatigue in people with IRCs [43, 44]. Fatigue may also be impacted by the emotional and social aspects of the disease and joint inflammation [45].

However, the majority of evidence found no improvements in pain or disease activity. This is surprising as the overwhelming consensus in the literature is that increased levels of anxiety and depression are associated with increased disease activity and pain [8, 46–49]. The apparent disparity may be due to the disease activity measures used as only three of them were patient-reported indices (e.g. BASDAI, SLEDAI), with the rest using ESR or CRP levels which would not be expected to change, whereas patient global or tender joint count may change with mood.

This review synthesizes the evidence around the use of psychosocial interventions to prevent anxiety and depression in IRCs and highlights that some interventions could be considered but there is a need for these to be more robustly evaluated. Given the impact of depression and anxiety on people with IRCs, such evaluation of effectiveness and acceptability is warranted.

## Supplementary Material

rkag059_Supplementary_Data

## Data Availability

The data used and analysed during the systematic review are available from the corresponding author on reasonable request.
