# Supplementary material for: Impact of interventions to prevent anxiety and depression in people with inflammatory rheumatological conditions: a systematic review
Source: Rheumatol Adv Pract. 2026 May 29;10(3):rkag059. doi: 10.1093/rap/rkag059 (PMC13268797; doi:10.1093/rap/rkag059)
Supplement: rkag059_Supplementary_Data [file rkag059_supplementary_data.zip › Supplementary_Table_2_GRADE_Criteria.docx]

Supplementary Table S2: GRADE Criteria

| **Factors that may lead to downgrading** |  |
| --- | --- |
| Number of studies | Concern if under 3 or under studies |
| Risk of bias | Concern if 50%≤ have high or unclear risk of bias |
| Inconsistency | Concern if 50%≥ do not show significant improvement |
| Imprecision | Concern if 50%≤ have under 50 participants per arm |
| **Factors that may lead to upgrading** |  |
| Magnitude | Upgraded if effect size is large |
|  |  |
