# Supplementary material for: Impact of interventions to prevent anxiety and depression in people with inflammatory rheumatological conditions: a systematic review
Source: Rheumatol Adv Pract. 2026 May 29;10(3):rkag059. doi: 10.1093/rap/rkag059 (PMC13268797; doi:10.1093/rap/rkag059)
Supplement: rkag059_Supplementary_Data [file rkag059_supplementary_data.zip › Supplementary_Table_1_Database_Searches.docx]

Supplementary Table S1: Full Database Searches

| Ovid MEDLINE <1946 to October 1, 2024> |
| --- |
| 1 exp Chronic Disease/ |
| 2 (long term adj3 (condition* or disease* or disorder* or illness*)).ti,ab,kf. |
| 3 (longterm adj3 (condition* or disease* or disorder* or illness*)).ti,ab,kf. |
| 4 (chronic* adj3 (condition* or disease* or disorder* or illness*)).ti,ab,kf. |
| 5 (longstanding adj3 (condition* or disease* or disorder* or illness*)).ti,ab,kf. |
| 6 (long standing adj3 (condition* or disease* or disorder* or illness*)).ti,ab,kf. |
| 7 LTC*.ti,ab,kf. |
| 8 Chronic.hw. |
| 9 rheumatic diseases/ or exp arthritis, rheumatoid/ or exp gout/ or polymyalgia rheumatica/ |
| 10 exp Lupus Erythematosus, Systemic/ |
| 11 exp Spondylarthropathies/ |
| 12 Giant Cell Arteritis/ |
| 13 (rheumat* adj3 (arthrit* or disease* or condition* or nodule*)).ti,ab,kf. |
| 14 (Inflamm* adj3 (arthrit* or rheumat*)).ti,ab,kf. |
| 15 ankylosing spondylitis.ti,ab,kf. |
| 16 psoriatic arthritis.ti,ab,kf. |
| 17 polymyalgia.ti,ab,kf. |
| 18 arthropath*.ti,ab,kf. |
| 19 arthrosis.ti,ab,kf. |
| 20 arthroses.ti,ab,kf. |
| 21 Gout*.ti,ab,kf. |
| 22 polyarthropath*.ti,ab,kf. |
| 23 polyarthriti*.ti,ab,kf. |
| 24 PMR*.ti,ab,kf. |
| 25 pPsA.ti,ab,kf. |
| 26 axPsA.ti,ab,kf. |
| 27 systemic lupus erythematosus.ti,ab,kf. |
| 28 axial spondy*.ti,ab,kf. |
| 29 AxSpA.ti,ab,kf. |
| 30 GCA*.ti,ab,kf. |
| 31 exp Osteoporosis/ |
| 32 osteoporo*.ti,ab,kf. |
| 33 exp Diabetes Mellitus/ |
| 34 diabet*.ti,ab,kf. |
| 35 exp Coronary Disease/ |
| 36 (coronary adj3 (condition* or disease* or disorder* or illness* or event*)).ti,ab,kf. |
| 37 exp Heart Diseases/ |
| 38 (cardiac* adj3 (condition* or disease* or disorder* or illness* or event* or arrest or arrhythmia*)).ti,ab,kf. |
| 39 atrial fibrillation.ti,ab,kf. |
| 40 exp Heart Failure/ |
| 41 (heart adj3 (attack* or condition* or disease* or disorder* or fail* or insuff*)).ti,ab,kf. |
| 42 (myocard* adj3 infarction).ti,ab,kf. |
| 43 exp Peripheral Arterial Disease/ |
| 44 peripheral arterial disease.ti,ab,kf. |
| 45 exp Stroke/ |
| 46 Stroke*1.ti,ab,kf. |
| 47 ((brain or cereb*) adj3 (ish?emia or infarct*)).ti,ab,kf. |
| 48 (cerebrovascular adj3 accident).ti,ab,kf. |
| 49 (acute adj3 cerebrovascular).ti,ab,kf. |
| 50 CVA*.ti,ab,kf. |
| 51 cerebral infarct*.ti,ab,kf. |
| 52 Ischemic Attack, Transient/ |
| 53 TIA*1.ti,ab,kf. |
| 54 Transient ischemic.ti,ab,kf. |
| 55 exp Renal Insufficiency, Chronic/ |
| 56 (kidney adj3 (insufficien* or fail*)).ti,ab,kf. |
| 57 (renal adj3 (insufficien* or fail*)).ti,ab,kf. |
| 58 (Chronic adj3 (kidney or renal)).ti,ab,kf. |
| 59 CKD*.ti,ab,kf. |
| 60 exp Pulmonary Disease, Chronic Obstructive/ |
| 61 emphysema*.ti,ab,kf. |
| 62 (chronic* adj3 bronchiti*).ti,ab,kf. |
| 63 COPD.ti,ab,kf. |
| 64 COAD.ti,ab,kf. |
| 65 exp Asthma/ |
| 66 asthma*.ti,ab,kf. |
| 67 exp Epilepsy/ |
| 68 exp Seizures/ |
| 69 (epilep*or seizure* or convuls*).ti,ab,kf. |
| 70 or/1-69 |
| 71 Depression/ |
| 72 exp Mood Disorders/ |
| 73 Dysthymic Disorder/ |
| 74 Anxiety/ |
| 75 exp Anxiety Disorders/ |
| 76 or/72-75 |
| 77 pc.fs. |
| 78 prevent*.ti,ab,kf. |
| 79 77 or 78 |
| 80 76 and 79 |
| 81 preventive health services/ or primary prevention/ or secondary prevention/ |
| 82 (anxi* or depress*).ti,ab,kf. |
| 83 76 or 82 |
| 84 81 and 83 |
| 85 80 or 84 |
| 86 (depress* adj3 (reduc* or first episode* or onset* or prevent* or risk or at-risk or symptom*)).ti,ab,kf. |
| 87 (dysthymi* adj3 (reduc* or first episode* or onset* or prevent* or risk or at-risk or symptom*)).ti,ab,kf. |
| 88 (affective disorder* adj3 (reduc* or first episode* or onset* or prevent* or risk or at-risk or symptom*)).ti,ab,kf. |
| 89 (melanchol* adj3 (reduc* or first episode* or onset* or prevent* or risk or at-risk or symptom*)).ti,ab,kf. |
| 90 (mood adj3 (reduc* or first episode* or onset* or prevent* or risk or at-risk or symptom*)).ti,ab,kf. |
| 91 (depress* adj3 (subclinical* or sub-clinical* or subthreshold* or sub-threshold* or subsyndrom* or sub-syndrom*)).ti,ab,kf. |
| 92 ((Mild* or minor) adj3 depress*).ti,ab,kf. |
| 93 low mood.ti,ab,kf. |
| 94 depress*.ti. and ("Quality of Life"/ or adaptation, psychological/ or psychosocial intervention/) |
| 95 (anxi* adj3 (reduc* or first episode* or onset* or prevent* or risk or at-risk or symptom*)).ti,ab,kf. |
| 96 (panic* adj3 (reduc* or first episode* or onset* or prevent* or risk or at-risk or symptom*)).ti,ab,kf. |
| 97 (stress* adj3 (reduc* or first episode* or onset* or prevent* or risk or at-risk or symptom*)).ti,ab,kf. |
| 98 (anxi* adj3 (subclinical* or sub-clinical* or subthreshold* or sub-threshold* or subsyndrom* or sub-syndrom*)).ti,ab,kf. |
| 99 anxi*.ti. and ("Quality of Life"/ or adaptation, psychological/ or psychosocial intervention/) |
| 100 (Anxi* or depress*).ti. and prevent*.ti,ab,kf. |
| 101 or/85-100 |
| 102 randomized controlled trial.pt. |
| 103 controlled clinical trial.pt. |
| 104 randomi?ed.ab. |
| 105 placebo.ab. |
| 106 clinical trials as topic.sh. |
| 107 randomly.ab. |
| 108 trial.ti. |
| 109 or/102-108 [Cochrane RCT Filter: sensitivity- and precision-maximizing version (2008 revision)] |
| 110 70 and 101 and 109 [RCTs of all LTCs] |
| 111 exp Cohort Studies/ |
| 112 controlled before-after studies/ |
| 113 historically controlled study/ |
| 114 interrupted time series analysis/ |
| 115 cohort.ti,ab,kf. |
| 116 (before adj2 after).ti,ab,kf. |
| 117 (pre adj2 post).ti,ab,kf. |
| 118 (pretest adj2 posttest).ti,ab,kf. |
| 119 (pre test adj2 post test).ti,ab,kf. |
| 120 control*.ti,ab,kf. |
| 121 time series.ti,ab,kf. |
| 122 or/111-121 [Controlled studies (not RCTs)] |
| 123 or/9-30 [all IRCs] |
| 124 123 and 101 and 122 [controlled studies of IRCs] |
| 125 110 or 124 |
| 126 exp animals/ not humans.sh. |
| 127 125 not 126 [RCTs of LTCs + Controlled studies of IRCs] |

**Ovid MEDLINE <1946 to October 1, 2024>**

| 1 | exp chronic disease/ |
| --- | --- |
| 2 | (long term adj3 (condition* or disease* or disorder* or illness*)).ti,ab,kf. |
| 3 | (longterm adj3 (condition* or disease* or disorder* or illness*)).ti,ab,kf. |
| 4 | (chronic* adj3 (condition* or disease* or disorder* or illness*)).ti,ab,kf. |
| 5 | (longstanding adj3 (condition* or disease* or disorder* or illness*)).ti,ab,kf. |
| 6 | (long standing adj3 (condition* or disease* or disorder* or illness*)).ti,ab,kf. |
| 7 | LTC*.ti,ab,kf. |
| 8 | Chronic.hw. |
| 9 | rheumatic disease/ or exp rheumatoid arthritis/ or exp gout/ or rheumatic polymyalgia/ |
| 10 | exp systemic lupus erythematosus/ |
| 11 | exp spondyloarthropathy/ |
| 12 | ankylosing spondylitis/ |
| 13 | giant cell arteritis/ |
| 14 | (rheumat* adj3 (arthrit* or disease* or condition* or nodule*)).ti,ab,kf. |
| 15 | (Inflamm* adj3 (arthrit* or rheumat*)).ti,ab,kf. |
| 16 | ankylosing spondylitis.ti,ab,kf. |
| 17 | psoriatic arthritis.ti,ab,kf. |
| 18 | polymyalgia.ti,ab,kf. |
| 19 | arthropath*.ti,ab,kf. |
| 20 | arthrosis.ti,ab,kf. |
| 21 | arthroses.ti,ab,kf. |
| 22 | Gout*.ti,ab,kf. |
| 23 | polyarthropath*.ti,ab,kf. |
| 24 | polyarthriti*.ti,ab,kf. |
| 25 | PMR*.ti,ab,kf. |
| 26 | pPsA.ti,ab,kf. |
| 27 | axPsA.ti,ab,kf. |
| 28 | systemic lupus erythematosus.ti,ab,kf. |
| 29 | axial spondy*.ti,ab,kf. |
| 30 | AxSpA.ti,ab,kf. |
| 31 | GCA*.ti,ab,kf. |
| 32 | exp osteoporosis/ |
| 33 | osteoporo*.ti,ab,kf. |
| 34 | exp diabetes mellitus/ |
| 35 | diabet*.ti,ab,kf. |
| 36 | exp coronary artery disease/ |
| 37 | (coronary adj3 (condition* or disease* or disorder* or illness* or event*)).ti,ab,kf. |
| 38 | exp heart disease/ |
| 39 | (cardiac* adj3 (condition* or disease* or disorder* or illness* or event* or arrest or arrhythmia*)).ti,ab,kf. |
| 40 | atrial fibrillation.ti,ab,kf. |
| 41 | exp heart failure/ |
| 42 | (heart adj3 (attack* or condition* or disease* or disorder* or fail* or insuff*)).ti,ab,kf. |
| 43 | (myocard* adj3 infarction).ti,ab,kf. |
| 44 | exp peripheral occlusive artery disease/ |
| 45 | peripheral arterial disease.ti,ab,kf. |
| 46 | exp cerebrovascular accident/ |
| 47 | Stroke*1.ti,ab,kf. |
| 48 | ((brain or cereb*) adj3 (ish?emia or infarct*)).ti,ab,kf. |
| 49 | (cerebrovascular adj3 accident).ti,ab,kf. |
| 50 | (acute adj3 cerebrovascular).ti,ab,kf. |
| 51 | CVA*.ti,ab,kf. |
| 52 | cerebral infarct*.ti,ab,kf. |
| 53 | transient ischemic attack/ |
| 54 | TIA*1.ti,ab,kf. |
| 55 | Transient ischemic.ti,ab,kf. |
| 56 | exp chronic kidney failure/ |
| 57 | (kidney adj3 (insufficien* or fail*)).ti,ab,kf. |
| 58 | (renal adj3 (insufficien* or fail*)).ti,ab,kf. |
| 59 | (Chronic adj3 (kidney or renal)).ti,ab,kf. |
| 60 | CKD*.ti,ab,kf. |
| 61 | exp chronic obstructive lung disease/ |
| 62 | emphysema*.ti,ab,kf. |
| 63 | (chronic* adj3 bronchiti*).ti,ab,kf. |
| 64 | COPD.ti,ab,kf. |
| 65 | COAD.ti,ab,kf. |
| 66 | exp asthma/ |
| 67 | asthma*.ti,ab,kf. |
| 68 | exp epilepsy/ |
| 69 | exp seizure/ |
| 70 | (epilep*or seizure* or convuls*).ti,ab,kf. |
| 71 | 1 or 2 or 3 or 4 or 5 or 6 or 7 or 8 or 9 or 10 or 11 or 12 or 13 or 14 or 15 or 16 or 17 or 18 or 19 or 20 or 21 or 22 or 23 or 24 or 25 or 26 or 27 or 28 or 29 or 30 or 31 or 32 or 33 or 34 or 35 or 36 or 37 or 38 or 39 or 40 or 41 or 42 or 43 or 44 or 45 or 46 or 47 or 48 or 49 or 50 or 51 or 52 or 53 or 54 or 55 or 56 or 57 or 58 or 59 or 60 or 61 or 62 or 63 or 64 or 65 or 66 or 67 or 68 or 69 or 70 |
| 72 | exp depression/ |
| 73 | mood disorder/ |
| 74 | anxiety/ |
| 75 | exp anxiety disorder/ |
| 76 | 72 or 73 or 74 or 75 |
| 77 | pc.fs. |
| 78 | prevent*.ti,ab,kf. |
| 79 | 77 or 78 |
| 80 | 76 and 79 |
| 81 | preventive health service/ or primary prevention/ or secondary prevention/ or prevention/ |
| 82 | (anxi* or depress*).ti,ab,kf. |
| 83 | 76 or 82 |
| 84 | 81 and 83 |
| 85 | 80 or 84 |
| 86 | (depress* adj3 (reduc* or first episode* or onset* or prevent* or risk or at-risk or symptom*)).ti,ab,kf. |
| 87 | (dysthymi* adj3 (reduc* or first episode* or onset* or prevent* or risk or at-risk or symptom*)).ti,ab,kf. |
| 88 | (affective disorder* adj3 (reduc* or first episode* or onset* or prevent* or risk or at-risk or symptom*)).ti,ab,kf. |
| 89 | (melanchol* adj3 (reduc* or first episode* or onset* or prevent* or risk or at-risk or symptom*)).ti,ab,kf. |
| 90 | (mood adj3 (reduc* or first episode* or onset* or prevent* or risk or at-risk or symptom*)).ti,ab,kf. |
| 91 | (depress* adj3 (subclinical* or sub-clinical* or subthreshold* or sub-threshold* or subsyndrom* or sub-syndrom*)).ti,ab,kf. |
| 92 | ((Mild* or minor) adj3 depress*).ti,ab,kf. |
| 93 | low mood.ti,ab,kf. |
| 94 | depress*.ti. and (exp "Quality of Life"/ or psychological adjustment/ or psychosocial intervention/) |
| 95 | (anxi* adj3 (reduc* or first episode* or onset* or prevent* or risk or at-risk or symptom*)).ti,ab,kf. |
| 96 | (panic* adj3 (reduc* or first episode* or onset* or prevent* or risk or at-risk or symptom*)).ti,ab,kf. |
| 97 | (stress* adj3 (reduc* or first episode* or onset* or prevent* or risk or at-risk or symptom*)).ti,ab,kf. |
| 98 | (anxi* adj3 (subclinical* or sub-clinical* or subthreshold* or sub-threshold* or subsyndrom* or sub-syndrom*)).ti,ab,kf. |
| 99 | anxi*.ti. and (exp "Quality of Life"/ or psychological adjustment/ or psychosocial intervention/) |
| 100 | (Anxi* or depress*).ti. and prevent*.ti,ab,kf. |
| 101 | 85 or 86 or 87 or 88 or 89 or 90 or 91 or 92 or 93 or 94 or 95 or 96 or 97 or 98 or 99 or 100 |
| 102 | random*.tw. |
| 103 | factorial*.tw. |
| 104 | crossover*.tw. |
| 105 | cross-over*.tw. |
| 106 | placebo*.tw. |
| 107 | (doubl* adj blind*).tw. |
| 108 | (singl* adj blind*).tw. |
| 109 | assign*.tw. |
| 110 | allocat*.tw. |
| 111 | volunteer*.tw. |
| 112 | crossover procedure/ |
| 113 | double blind procedure/ |
| 114 | randomized controlled trial/ |
| 115 | single blind procedure/ |
| 116 | 102 or 103 or 104 or 105 or 106 or 107 or 108 or 109 or 110 or 111 or 112 or 113 or 114 or 115 |
| 117 | 71 and 101 and 116 [RCTs of all LTCs] |
| 118 | control group/ |
| 119 | time series analysis/ |
| 120 | cohort analysis/ |
| 121 | control*.ti,ab,kf. |
| 122 | exp controlled study/ |
| 123 | (before adj2 after).ti,ab,kf. |
| 124 | (pre adj2 post).ti,ab,kf. |
| 125 | (pretest adj2 posttest).ti,ab,kf. |
| 126 | (pre test adj2 post test).ti,ab,kf. |
| 127 | time series.ti,ab,kf. |
| 128 | cohort.ti,ab,kf. |
| 129 | 118 or 119 or 120 or 121 or 122 or 123 or 124 or 125 or 126 or 127 or 128 [Controlled studies (not RCTs)] |
| 130 | 9 or 10 or 11 or 12 or 13 or 14 or 15 or 16 or 17 or 18 or 19 or 20 or 21 or 22 or 23 or 24 or 25 or 26 or 27 or 28 or 29 or 30 or 31 [all IRCs] |
| 131 | 101 and 129 and 130 [controlled studies of IRCs] |
| 132 | 117 or 131 |
| 133 | exp animal/ not human/ |
| 134 | 132 not 133 |
| 135 | limit 134 to embase |

**Ovid AMED <1946 to October 1, 2024>**

| 1 | exp Chronic disease/ |
| --- | --- |
| 2 | (long term adj3 (condition* or disease* or disorder* or illness*)).ti,ab. |
| 3 | (longterm adj3 (condition* or disease* or disorder* or illness*)).ti,ab. |
| 4 | (chronic* adj3 (condition* or disease* or disorder* or illness*)).ti,ab. |
| 5 | (longstanding adj3 (condition* or disease* or disorder* or illness*)).ti,ab. |
| 6 | (long standing adj3 (condition* or disease* or disorder* or illness*)).ti,ab. |
| 7 | LTC*.ti,ab. |
| 8 | Chronic.hw. |
| 9 | Rheumatic disease/ or exp Arthritis rheumatoid/ or exp Gout/ |
| 10 | exp Lupus erythematosus systemic/ |
| 11 | (rheumat* adj3 (arthrit* or disease* or condition* or nodule*)).ti,ab. |
| 12 | (Inflamm* adj3 (arthrit* or rheumat*)).ti,ab. |
| 13 | ankylosing spondylitis.ti,ab. |
| 14 | psoriatic arthritis.ti,ab. |
| 15 | polymyalgia.ti,ab. |
| 16 | arthropath*.ti,ab. |
| 17 | arthrosis.ti,ab. |
| 18 | arthroses.ti,ab. |
| 19 | Gout*.ti,ab. |
| 20 | polyarthriti*.ti,ab. |
| 21 | polyarthropath*.ti,ab. |
| 22 | PMR*.ti,ab. |
| 23 | systemic lupus erythematosus.ti,ab. |
| 24 | axial spondy*.ti,ab. |
| 25 | AxSpA.ti,ab. |
| 26 | GCA*.ti,ab. |
| 27 | exp Osteoporosis/ |
| 28 | osteoporo*.ti,ab. |
| 29 | exp Diabetes mellitus/ |
| 30 | diabet*.ti,ab. |
| 31 | (coronary adj3 (condition* or disease* or disorder* or illness* or event*)).ti,ab. |
| 32 | exp Heart disease/ |
| 33 | (cardiac* adj3 (condition* or disease* or disorder* or illness* or event* or arrest or arrhythmia*)).ti,ab. |
| 34 | atrial fibrillation.ti,ab. |
| 35 | (heart adj3 (attack* or condition* or disease* or disorder* or fail* or insuff*)).ti,ab. |
| 36 | (myocard* adj3 infarction).ti,ab. |
| 37 | exp peripheral vascular disease/ |
| 38 | peripheral arterial disease.ti,ab. |
| 39 | stroke/ |
| 40 | Stroke*1.ti,ab. |
| 41 | ((brain or cereb*) adj3 (ish?emia or infarct*)).ti,ab. |
| 42 | (cerebrovascular adj3 accident).ti,ab. |
| 43 | (acute adj3 cerebrovascular).ti,ab. |
| 44 | CVA*.ti,ab. |
| 45 | cerebral infarct*.ti,ab. |
| 46 | Cerebral ischemia/ |
| 47 | TIA*1.ti,ab. |
| 48 | Transient ischemic.ti,ab. |
| 49 | Kidney failure chronic/ |
| 50 | (kidney adj3 (insufficien* or fail*)).ti,ab. |
| 51 | (renal adj3 (insufficien* or fail*)).ti,ab. |
| 52 | (Chronic adj3 (kidney or renal)).ti,ab. |
| 53 | CKD*.ti,ab. |
| 54 | exp Pulmonary Disease Chronic Obstructive/ |
| 55 | emphysema*.ti,ab. |
| 56 | (chronic* adj3 bronchiti*).ti,ab. |
| 57 | COPD.ti,ab. |
| 58 | COAD.ti,ab. |
| 59 | exp Asthma/ |
| 60 | asthma*.ti,ab. |
| 61 | Epilepsy/ |
| 62 | exp Seizures/ |
| 63 | (epilep*or seizure* or convuls*).ti,ab. |
| 64 | 1 or 2 or 3 or 4 or 5 or 6 or 7 or 8 or 9 or 10 or 11 or 12 or 13 or 14 or 15 or 16 or 17 or 18 or 19 or 20 or 21 or 22 or 23 or 24 or 25 or 26 or 27 or 28 or 29 or 30 or 31 or 32 or 33 or 34 or 35 or 36 or 37 or 38 or 39 or 40 or 41 or 42 or 43 or 44 or 45 or 46 or 47 or 48 or 49 or 50 or 51 or 52 or 53 or 54 or 55 or 56 or 57 or 58 or 59 or 60 or 61 or 62 or 63 |
| 65 | Depression/ |
| 66 | depressive disorder/ |
| 67 | Anxiety/ |
| 68 | exp Anxiety disorders/ |
| 69 | 65 or 66 or 67 or 68 |
| 70 | prevent*.ti,ab. |
| 71 | 69 and 70 |
| 72 | Prevention/ |
| 73 | (anxi* or depress*).ti,ab. |
| 74 | 69 or 73 |
| 75 | 72 and 74 |
| 76 | 71 or 75 |
| 77 | (depress* adj3 (reduc* or first episode* or onset* or prevent* or risk or at-risk or symptom*)).ti,ab. |
| 78 | (dysthymi* adj3 (reduc* or first episode* or onset* or prevent* or risk or at-risk or symptom*)).ti,ab. |
| 79 | (affective disorder* adj3 (reduc* or first episode* or onset* or prevent* or risk or at-risk or symptom*)).ti,ab. |
| 80 | (melanchol* adj3 (reduc* or first episode* or onset* or prevent* or risk or at-risk or symptom*)).ti,ab. |
| 81 | (mood adj3 (reduc* or first episode* or onset* or prevent* or risk or at-risk or symptom*)).ti,ab. |
| 82 | (depress* adj3 (subclinical* or sub-clinical* or subthreshold* or sub-threshold* or subsyndrom* or sub-syndrom*)).ti,ab. |
| 83 | ((Mild* or minor) adj3 depress*).ti,ab. |
| 84 | low mood.ti,ab. |
| 85 | depress*.ti. and ("Quality of Life"/ or adaptation psychological/) |
| 86 | (anxi* adj3 (reduc* or first episode* or onset* or prevent* or risk or at-risk or symptom*)).ti,ab. |
| 87 | (panic* adj3 (reduc* or first episode* or onset* or prevent* or risk or at-risk or symptom*)).ti,ab. |
| 88 | (stress* adj3 (reduc* or first episode* or onset* or prevent* or risk or at-risk or symptom*)).ti,ab. |
| 89 | (anxi* adj3 (subclinical* or sub-clinical* or subthreshold* or sub-threshold* or subsyndrom* or sub-syndrom*)).ti,ab. |
| 90 | anxi*.ti. and ("Quality of Life"/ or adaptation psychological/) |
| 91 | (Anxi* or depress*).ti. and prevent*.ti,ab. |
| 92 | 76 or 77 or 78 or 79 or 80 or 81 or 82 or 83 or 84 or 85 or 86 or 87 or 88 or 89 or 90 or 91 |
| 93 | randomized controlled trials/ |
| 94 | single blind method/ |
| 95 | double blind method/ |
| 96 | random*.ti,ab. |
| 97 | factorial*.ti,ab. |
| 98 | (crossover* or cross over*).ti,ab. |
| 99 | placebo*.ti,ab. |
| 100 | (doub* adj blind*).ti,ab. |
| 101 | (sing* adj blind*).ti,ab. |
| 102 | assign*.ti,ab. |
| 103 | allocat*.ti,ab. |
| 104 | volunteer*.ti,ab. |
| 105 | 93 or 94 or 94 or 96 or 97 or 98 or 99 or 100 or 101 or 102 or 103 or 104 |
| 106 | 64 and 92 and 105 [RCTs of all LTCs] |
| 107 | exp cohort studies/ |
| 108 | cohort.ti,ab. |
| 109 | (pre adj2 post).ti,ab. |
| 110 | (pretest adj2 posttest).ti,ab. |
| 111 | (pre test adj2 post test).ti,ab. |
| 112 | control*.ti,ab. |
| 113 | time series.ti,ab. |
| 114 | 107 or 108 or 109 or 110 or 111 or 112 or 113 [controlled studies (not RCTs)] |
| 115 | 9 or 10 or 11 or 12 or 13 or 14 or 15 or 16 or 17 or 18 or 19 or 20 or 21 or 22 or 23 or 24 or 25 or 26 |
| 116 | 92 and 114 and 115 |
| 117 | 106 or 116 |
| 118 | exp animals/ not humans.sh. |
| 119 | 117 not 118 [RCTs of LTCs + Controlled studies of IRCs] |

**EBSCO CINAHL Plus <1946 to October 1, 2024>**

| S1 | (MH "Chronic Disease+") |
| --- | --- |
| S2 | MW chronic |
| S3 | TI ( ("long term" N2 (condition* or disease* or disorder* or illness*)) ) OR AB ( ("long term" N2 (condition* or disease* or disorder* or illness*)) |
| S4 | TI ( (longterm N2 (condition* or disease* or disorder* or illness*)) ) OR AB ( (longterm N2 (condition* or disease* or disorder* or illness*)) |
| S5 | TI ( (chronic* N2 (condition* or disease* or disorder* or illness*)) ) OR AB ( (chronic* N2 (condition* or disease* or disorder* or illness*)) |
| S6 | TI ( (longstanding N2 (condition* or disease* or disorder* or illness*)) ) OR AB ( (longstanding N2 (condition* or disease* or disorder* or illness*)) |
| S7 | TI ( ("long standing" N2 (condition* or disease* or disorder* or illness*)) ) OR AB ( ("long standing" N2 (condition* or disease* or disorder* or illness*)) |
| S8 | TI LTC* OR AB LTC* |
| S9 | (MH "Rheumatic Diseases") OR (MH "Arthritis, Rheumatoid+") OR (MH "Gout") OR (MH "Polymyalgia Rheumatica") |
| S10 | (MH "Lupus Erythematosus, Systemic+") |
| S11 | (MH "Spondylarthropathies+") |
| S12 | (MH "Giant Cell Arteritis") |
| S13 | TI ((rheumat* N2 (arthrit* or disease* or condition* or nodule*)) ) OR AB ((rheumat* N2 (arthrit* or disease* or condition* or nodule*)) |
| S14 | TI ((inflamm* N2 (arthrit* or rheumat*)) OR AB ((inflamm* N2 (arthrit* or rheumat*)) |
| S15 | TI "ankylosing spondylitis" OR AB "ankylosing spondylitis" |
| S16 | TI "psoriatic arthritis" OR AB "psoriatic arthritis" |
| S17 | TI polymyalgia OR AB polymyalgia |
| S18 | TI anthropath* OR AB anthropath* |
| S19 | TI arthrosis OR AB arthrosis |
| S20 | TI arthroses OR AB arthroses |
| S21 | TI gout* OR AB gout* |
| S22 | TI polyarthropath* OR AB polyarthropath* |
| S23 | TI polyarthriti* OR AB polyarthriti* |
| S24 | TI PMR* OR AB PMR* |
| S25 | TI pPsA OR AB pPsA |
| S26 | TI axPsA OR AB axPsA |
| S27 | TI "systemic lupus erythematosus" OR AB "systemic lupus erythematosus" |
| S28 | TI "axial spondy*" OR AB "axial spondy*" |
| S29 | TI AxSpA OR AB AxSpA |
| S30 | TI GCA* OR AB GCA* |
| S31 | (MH "Osteoporosis+") |
| S32 | TI osteoporo* OR AB osteoporo* |
| S33 | (MH "Diabetes Mellitus+") |
| S34 | TI diabet* OR AB diabet* |
| S35 | (MH "Coronary Disease+") |
| S36 | TI ((coronary N2 (condition* or disease* or disorder* or illness* or event*)) OR AB ((coronary N2 (condition* or disease* or disorder* or illness* or event*)) |
| S37 | (MH "Heart Diseases+") |
| S38 | TI ((cardiac* N2 (condition* or disease* or disorder* or illness* or event* or arrest or arrhythmia*)) OR AB ((cardiac* N2 (condition* or disease* or disorder* or illness* or event* or arrest or arrhythmia)) |
| S39 | TI "atrial fibrillation" OR AB "atrial fibrillation |
| S40 | (MH "Heart Failure+") |
| S41 | TI ((heart N2 (attack* or condition* or disease* or disorder* or fail* or insuff*)) OR AB ((heart N2 (attack* or condition* or disease* or disorder* or fail* or insuff*)) |
| S42 | TI (myocard* N2 infarction) OR AB (myocard* N2 infarction) |
| S43 | (MH "Peripheral Vascular Diseases+") |
| S44 | TI "peripheral arterial disease" OR AB "peripheral arterial disease" |
| S45 | (MH "Stroke+") |
| S46 | TI Stroke* OR AB Stroke* |
| S47 | TI ((brain or cereb*) N2 (ish?emia or infarct*)) OR AB ((brain or cereb*) N2 (ish?emia or infarct*)) |
| S48 | TI (acute N2 cerebrovascular) OR AB (acute N2 cerebrovascular) |
| S49 | TI (cerebrovascular N2 accident) OR AB (cerebrovascular N2 accident) |
| S50 | TI CVA* OR AB CVA* |
| S51 | TI "cerebral infarct*" OR AB "cerbral infarct*" |
| S52 | (MH "Cerebral Ischemia, Transient") |
| S53 | TI TIA* OR AB TIA* |
| S54 | TI "transient ischemic" OR AB "transient ischemic" |
| S55 | (MH "Renal Insufficiency, Chronic+") |
| S56 | TI (kidney N2 (insufficien* or fail*)) OR AB (kidney N2 (insufficien* or fail*)) |
| S57 | TI (renal N2 (insufficien* or fail*)) OR AB (renal N2 (insufficien* or fail*)) |
| S58 | TI (chronic N2 (kidney or renal)) OR AB (chornic N2 (kidney or renal)) |
| S59 | TI CKD* OR AB CKD* |
| S60 | (MH "Pulmonary Disease, Chronic Obstructive+") |
| S61 | TI emphysema* OR AB emphysema* |
| S62 | TI (chronic* N2 bronchiti*) OR AB (chronic* N2 bronchiti*) |
| S63 | TI COPD* OR AB COPD* |
| S64 | TI COAD* OR AB COAD* |
| S65 | (MH "Asthma+") |
| S66 | TI asthma* OR AB asthma* |
| S67 | (MH "Epilepsy+") |
| S68 | (MH "Seizures+") |
| S69 | TI (epilep* or seizure* or convuls*) OR AB (epilep* or seizure* or convuls*) |
| S70 | S1 OR S2 OR S3 OR S4 OR S5 OR S6 OR S7 OR S8 OR S9 OR S10 OR S11 OR S12 OR S13 OR S14 OR S15 OR S16 OR S17 OR S18 OR S19 OR S20 OR S21 OR S22 OR S23 OR S24 OR S25 OR S26 OR S27 OR S28 OR S29 OR S30 OR S31 OR S32 OR S33 OR S34 OR S35 OR S36 OR S37 OR S38 OR S39 OR S40 OR S41 OR S42 OR S43 OR S44 OR S45 OR S46 OR S47 OR S48 OR S49 OR S50 OR S51 OR S52 OR S53 OR S54 OR S55 OR S56 OR S57 OR S58 OR S59 OR S60 OR S61 OR S62 OR S63 OR S64 OR S65 OR S66 OR S67 OR S68 OR S69 |
| S71 | (MH "Depression+") |
| S72 | (MH "Affective Disorders") |
| S73 | (MH "Anxiety") |
| S74 | (MH "Anxiety Disorders+") |
| S75 | S71 OR S72 OR S73 OR S74 |
| S76 | MW "pc" |
| S77 | TI prevent* OR AB prevent* |
| S78 | S76 OR S77 |
| S79 | S75 AND S78 |
| S80 | (MH "Preventive Health Care") |
| S81 | TI (anxi* or depress*) OR AB (anxi* or depress*) |
| S82 | S75 OR S81 |
| S83 | S80 AND S82 |
| S84 | S79 OR S83 |
| S85 | TI (depress* N2 (reduc* or first episode* or onset* or prevent* or risk or at-risk or symptom*)) OR AB (depress* N2 (reduc* or first episode* or onset* or prevent* or risk or at-risk or symptom*)) |
| S86 | TI (dysthymi* N2 (reduc* or first episode* or onset* or prevent* or risk or at-risk or symptom*)) OR AB (dysthymi* N2 (reduc* or first episode* or onset* or prevent* or risk or at-risk or symptom*)) |
| S87 | TI (affective disorder* N2 (reduc* or first episode* or onset* or prevent* or risk or at-risk or symptom*)) (affective disorder* N2 (reduc* or first episode* or onset* or prevent* or risk or at-risk or symptom*)) OR AB (affective disorder* N2 (reduc* or first episode* or onset* or prevent* or risk or at-risk or symptom*)) |
| S88 | TI (melanchol* N2 (reduc* or first episode* or onset* or prevent* or risk or at-risk or symptom*)) (melanchol* N2 (reduc* or first episode* or onset* or prevent* or risk or at-risk or symptom*)) OR AB (affective disorder* N2 (reduc* or first episode* or onset* or prevent* or risk or at-risk or symptom*)) |
| S89 | TI (mood N2 (reduc* or first episode* or onset* or prevent* or risk or at-risk or symptom*)) (mood N2 (reduc* or first episode* or onset* or prevent* or risk or at-risk or symptom*)) OR AB (affective disorder* N2 (reduc* or first episode* or onset* or prevent* or risk or at-risk or symptom*)) |
| S90 | TI (depress* N2 (subclinical* or sub-clinical* or subthreshold* or sub-threshold* or subsyndrom* or sub-syndrom*)) OR AB (depress* N2 (subclinical* or sub-clinical* or subthreshold* or sub-threshold* or subsyndrom* or sub-syndrom*)) |
| S91 | TI ((Mild* or minor) N2 depress*) OR AB ((Mild* or minor) N2 depress*) |
| S92 | TI "low mood" OR AB "low mood" |
| S93 | TI depress* AND ((MH "Quality of Life") OR (MH "Quality of Working Life") OR (MH "Adaptation, Psychological") OR (MH "Psychosocial Intervention")) |
| S94 | TI (anxi* N2 (reduc* or first episode* or onset* or prevent* or risk or at-risk or symptom*)) OR AB (anxi* N2 (reduc* or first episode* or onset* or prevent* or risk or at-risk or symptom*)) |
| S95 | TI (panic* N2 (reduc* or first episode* or onset* or prevent* or risk or at-risk or symptom*)) OR AB (panic* N2 (reduc* or first episode* or onset* or prevent* or risk or at-risk or symptom*)) |
| S96 | TI (stress* N2 (reduc* or first episode* or onset* or prevent* or risk or at-risk or symptom*)) OR AB (stress* N2 (reduc* or first episode* or onset* or prevent* or risk or at-risk or symptom*)) |
| S97 | TI (anxi* N2 (subclinical* or sub-clinical* or subthreshold* or sub-threshold* or subsyndrom* or sub-syndrom*)) OR AB (anxi* N2 (subclinical* or sub-clinical* or subthreshold* or sub-threshold* or subsyndrom* or sub-syndrom*)) |
| S98 | TI anxi* AND ((MH "Quality of Life") OR (MH "Adaptation, Psychological") OR (MH "Psychosocial intervention")) |
| S99 | TI anxi* AND TI (prevent* N2 (primary or selective or indicated)) OR AB (prevent* N2 (primary or selective or indicated)) |
| S100 | S84 OR S85 OR S86 OR S87 OR S88 OR S89 OR S90 OR S91 OR S92 OR S93 OR S94 OR S95 OR S96 OR S97 OR S98 OR S99 |
| S101 | (MH "Clinical Trials+") |
| S102 | TI random* OR AB random* |
| S103 | TI crossover* OR AB crossover* |
| S104 | TI cross W0 over* OR AB cross W0 over* |
| S105 | TI placebo* OR AB placebo* |
| S106 | TI doubl* W0 blind* OR AB doubl* W0 blind* |
| S107 | TI singl* W0 blind* OR AB singl* W0 blind* |
| S108 | TI assign* OR AB assign* |
| S109 | TI allocat* OR AB allocat* |
| S110 | TI volunteer* OR AB volunteer* |
| S111 | S101 OR S102 OR S103 OR S104 OR S105 OR S106 OR S107 OR S108 OR S109 OR S110 |
| S112 | S70 AND S100 AND S111 |
| S113 | TI before N1 after OR AB before N1 after |
| S114 | TI pre N1 post OR AB pre N1 post |
| S115 | TI pretest N1 posttest OR AB pretest N1 posttest |
| S116 | TI "pre test" N1 "post test" OR AB "pre test" N1 "post test" |
| S117 | TI control* OR AB control* |
| S118 | TI ( "time series" OR cohort ) OR ( AB "time series" OR cohort) |
| S119 | S113 OR S114 OR S115 OR S116 OR S117 OR S118 |
| S120 | S9 OR S10 OR S11 OR S12 OR S13 OR S14 OR S15 OR S16 OR S17 OR S18 OR S19 OR S20 OR S21 OR S22 OR S23 OR S24 OR S25 OR S26 OR S27 OR S28 OR S29 OR S30 |
| S121 | S100 AND S119 AND S120 |
| S122 | S112 OR S121 |

**EBSCO APAPSYCInfo <1946 to October 1, 2024>**

| S1 | DE "Chronic Illness" OR DE "Chronic Pain" OR DE "Chronic Symptoms" |
| --- | --- |
| S2 | MJ chronic |
| S3 | TI ( ("long term" N2 (condition* or disease* or disorder* or illness*)) ) OR AB ( ("long term" N2 (condition* or disease* or disorder* or illness*)) ) |
| S4 | TI ( (longterm N2 (condition* or disease* or disorder* or illness*)) ) OR AB ( (longterm N2 (condition* or disease* or disorder* or illness*)) ) |
| S5 | TI ( (chronic* N2 (condition* or disease* or disorder* or illness*)) ) OR AB ( (chronic* N2 (condition* or disease* or disorder* or illness*)) ) |
| S6 | TI ( (longstanding N2 (condition* or disease* or disorder* or illness*)) ) OR AB ( (longstanding N2 (condition* or disease* or disorder* or illness*)) ) |
| S7 | TI ( ("long standing" N2 (condition* or disease* or disorder* or illness*)) ) OR AB ( ("long standing" N2 (condition* or disease* or disorder* or illness*)) ) |
| S8 | TI LTC* OR AB LTC* |
| S9 | DE "Rheumatoid Arthritis" |
| S10 | TI ((rheumat* N2 (arthrit* or disease* or condition* or nodule*)) ) OR AB ((rheumat* N2 (arthrit* or disease* or condition* or nodule*)) ) |
| S11 | TI ((inflamm* N2 (arthrit* or rheumat*)) OR AB ((inflamm* N2 (arthrit* or rheumat*)) |
| S12 | TI "ankylosing spondylitis" OR AB "ankylosing spondylitis" |
| S13 | TI "psoriatic arthritis" OR AB "psoriatic arthritis" |
| S14 | TI polymyalgia OR AB polymyalgia |
| S15 | TI anthropath* OR AB anthropath* |
| S16 | TI arthrosis OR AB arthrosis |
| S17 | TI arthroses OR AB arthroses |
| S18 | TI gout* OR AB gout* |
| S19 | TI polyarthropath* OR AB polyarthropath* |
| S20 | TI polyarthriti* OR AB polyarthriti* |
| S21 | TI PMR* OR AB PMR* |
| S22 | TI pPsA OR AB pPsA |
| S23 | TI axPsA OR AB axPsA |
| S24 | TI "systemic lupus erythematosus" OR AB "systemic lupus erythematosus" |
| S25 | TI "axial spondy*" OR AB "axial spondy*" |
| S26 | TI AxSpA OR AB AxSpA |
| S27 | TI GCA* OR AB GCA* |
| S28 | DE "Osteoporosis" |
| S29 | TI osteoporo* OR AB osteoporo* |
| S30 | DE "Diabetes Mellitus" or DE "Type 2 Diabetes" |
| S31 | TI diabet* OR AB diabet* |
| S32 | DE "Heart Disorders" OR DE "Angina Pectoris" OR DE "Arrhythmias (Heart)" OR DE "Coronary Heart Disease" OR DE "Coronary Thromboses" OR DE "Myocardial Infarctions" |
| S33 | TI ((coronary N2 (condition* or disease* or disorder* or illness* or event*)) OR AB ((coronary N2 (condition* or disease* or disorder* or illness* or event*)) ) |
| S34 | TI ((cardiac* N2 (condition* or disease* or disorder* or illness* or event* or arrest or arrhythmia*)) OR AB ((cardiac* N2 (condition* or disease* or disorder* or illness* or event* or arrest or arrhythmia)) ) |
| S35 | TI "atrial fibrillation" OR AB "atrial fibrillation" |
| S36 | TI ((heart N2 (attack* or condition* or disease* or disorder* or fail* or insuff*)) OR AB ((heart N2 (attack* or condition* or disease* or disorder* or fail* or insuff*)) |
| S37 | TI (myocard* N2 infarction) OR AB (myocard* N2 infarction) |
| S38 | TI "peripheral arterial disease" OR AB "peripheral arterial disease" |
| S39 | DE "Cerebrovascular Accidents" OR DE "Cerebrovascular Disorders" OR DE "Cerebral Arteriosclerosis" OR DE "Cerebral Hemorrhage" OR DE "Cerebral Ischemia" OR DE "Cerebral Small Vessel Disease" OR DE "Cerebrovascular Accidents" OR DE "Subarachnoid Hemorrhage" |
| S40 | TI Stroke* OR AB Stroke* |
| S41 | TI ((brain or cereb*) N2 (ish?emia or infarct*)) OR AB ((brain or cereb*) N2 (ish?emia or infarct*)) |
| S42 | TI (acute N2 cerebrovascular) OR AB (acute N2 cerebrovascular) |
| S43 | TI CVA* OR AB CVA* |
| S44 | TI "cerebral infarct*" OR AB "cerbral infarct*" |
| S45 | TI TIA* OR AB TIA* |
| S46 | TI "transient ischemic" OR AB "transient ischemic" |
| S47 | DE "Kidney Diseases" |
| S48 | TI (kidney N2 (insufficien* or fail*)) OR AB (kidney N2 (insufficien* or fail*)) |
| S49 | TI (renal N2 (insufficien* or fail*)) OR AB (renal N2 (insufficien* or fail*)) |
| S50 | TI (chronic N2 (kidney or renal)) OR AB (chornic N2 (kidney or renal)) |
| S51 | TI CKD* OR AB CKD* |
| S52 | DE "Chronic Obstructive Pulmonary Disease" OR DE "Bronchial Disorders" OR DE "Pulmonary Emphysema" |
| S53 | TI emphysema* OR AB emphysema* |
| S54 | TI (chronic* N2 bronchiti*) OR AB (chronic* N2 bronchiti*) |
| S55 | TI COPD* OR AB COPD* |
| S56 | TI COAD* OR AB COAD* |
| S57 | DE "Asthma" |
| S58 | TI asthma* OR AB asthma* |
| S59 | DE "Epilepsy" OR DE "Epileptic Seizures" OR DE "Lennox Gastaut Syndrome" |
| S60 | TI (epilep* or seizure* or convuls*) OR AB (epilep* or seizure* or convuls*) |
| S61 | S1 OR S2 OR S3 OR S4 OR S5 OR S6 OR S7 OR S8 OR S9 OR S10 OR S11 OR S12 OR S13 OR S14 OR S15 OR S16 OR S17 OR S18 OR S19 OR S20 OR S21 OR S22 OR S23 OR S24 OR S25 OR S26 OR S27 OR S28 OR S29 OR S30 OR S31 OR S32 OR S33 OR S34 OR S35 OR S36 OR S37 OR S38 OR S39 OR S40 OR S41 OR S42 OR S43 OR S44 OR S45 OR S46 OR S47 OR S48 OR S49 OR S50 OR S51 OR S52 OR S53 OR S54 OR S55 OR S56 OR S57 OR S58 OR S59 OR S60 |
| S62 | DE "Depression (Emotion)" |
| S63 | DE "Major Depression" OR DE "Anaclitic Depression" OR DE "Dysthymic Disorder" OR DE "Endogenous Depression" OR DE "Late Life Depression" OR DE "Postpartum Depression" OR DE "Reactive Depression" OR DE "Recurrent Depression" OR DE "Treatment Resistant Depression" |
| S64 | DE "Affective Disorders" |
| S65 | DE "Anxiety" |
| S66 | DE "Anxiety Disorders" OR DE "Castration Anxiety" OR DE "Generalized Anxiety Disorder" OR DE "Obsessive Compulsive Disorder" OR DE "Panic Attack" OR DE "Panic Disorder" OR DE "Phobias" OR DE "Separation Anxiety Disorder" OR DE "Trichotillomania" |
| S67 | S62 OR S63 OR S64 OR S65 OR S66 |
| S68 | TI prevent* OR AB prevent* |
| S69 | S67 AND S68 |
| S70 | DE "Prevention" OR DE "Preventive Health Services" |
| S71 | TI (anxi* or depress*) OR AB (anxi* or depress*) |
| S72 | S67 OR S71 |
| S73 | S70 AND S72 |
| S74 | S69 OR S73 |
| S75 | TI (depress* N2 (reduc* or first episode* or onset* or prevent* or risk or at-risk or symptom*)) OR AB (depress* N2 (reduc* or first episode* or onset* or prevent* or risk or at-risk or symptom*)) |
| S76 | TI (dysthymi* N2 (reduc* or first episode* or onset* or prevent* or risk or at-risk or symptom*)) OR AB (dysthymi* N2 (reduc* or first episode* or onset* or prevent* or risk or at-risk or symptom*)) |
| S77 | TI (affective disorder* N2 (reduc* or first episode* or onset* or prevent* or risk or at-risk or symptom*)) (affective disorder* N2 (reduc* or first episode* or onset* or prevent* or risk or at-risk or symptom*)) OR AB (affective disorder* N2 (reduc* or first episode* or onset* or prevent* or risk or at-risk or symptom*)) |
| S78 | TI (melanchol* N2 (reduc* or first episode* or onset* or prevent* or risk or at-risk or symptom*)) (melanchol* N2 (reduc* or first episode* or onset* or prevent* or risk or at-risk or symptom*)) OR AB (affective disorder* N2 (reduc* or first episode* or onset* or prevent* or risk or at-risk or symptom*)) |
| S79 | TI (mood N2 (reduc* or first episode* or onset* or prevent* or risk or at-risk or symptom*)) (mood N2 (reduc* or first episode* or onset* or prevent* or risk or at-risk or symptom*)) OR AB (affective disorder* N2 (reduc* or first episode* or onset* or prevent* or risk or at-risk or symptom*)) |
| S80 | TI (depress* N2 (subclinical* or sub-clinical* or subthreshold* or sub-threshold* or subsyndrom* or sub-syndrom*)) OR AB (depress* N2 (subclinical* or sub-clinical* or subthreshold* or sub-threshold* or subsyndrom* or sub-syndrom*)) |
| S81 | TI ((Mild* or minor) N2 depress*) OR AB ((Mild* or minor) N2 depress*) |
| S82 | TI "low mood" OR AB "low mood" |
| S83 | TI depress* AND (DE "Quality of Life" OR DE "Health Related Quality of Life" OR DE "Quality of Work Life" OR DE "Emotional Adjustment") |
| S84 | TI (anxi* N2 (reduc* or first episode* or onset* or prevent* or risk or at-risk or symptom*)) OR AB (anxi* N2 (reduc* or first episode* or onset* or prevent* or risk or at-risk or symptom*)) |
| S85 | TI (panic* N2 (reduc* or first episode* or onset* or prevent* or risk or at-risk or symptom*)) OR AB (panic* N2 (reduc* or first episode* or onset* or prevent* or risk or at-risk or symptom*)) |
| S86 | TI (stress* N2 (reduc* or first episode* or onset* or prevent* or risk or at-risk or symptom*)) OR AB (stress* N2 (reduc* or first episode* or onset* or prevent* or risk or at-risk or symptom*)) |
| S87 | TI (anxi* N2 (subclinical* or sub-clinical* or subthreshold* or sub-threshold* or subsyndrom* or sub-syndrom*)) OR AB (anxi* N2 (subclinical* or sub-clinical* or subthreshold* or sub-threshold* or subsyndrom* or sub-syndrom*)) |
| S88 | TI anxi* AND (DE "Quality of Life" OR OR DE "Health Related Quality of Life" OR DE "Quality of Work Life" OR DE "Emotional Adjustment") |
| S89 | TI anxi* AND TI (prevent* N2 (primary or selective or indicated)) OR AB (prevent* N2 (primary or selective or indicated)) |
| S90 | S74 OR S75 OR S76 OR S77 OR S78 OR S79 OR S80 OR S81 OR S82 OR S83 OR S84 OR S85 OR S86 OR S87 OR S88 OR S89 |
| S91 | DE "Randomized Controlled Trials" OR DE "Clinical Trials" OR DE "Randomized Clinical Trials" |
| S92 | TI volunteer* OR AB volunteer* |
| S93 | TI allocat* OR AB allocat* |
| S94 | TI assign* OR AB assign* |
| S95 | TI singl* W0 blind* OR AB singl* W0 blind* |
| S96 | TI doubl* W0 blind* OR AB doubl* W0 blind* |
| S97 | TI placebo* OR AB placebo* |
| S98 | TI cross W0 over* OR AB cross W0 over* |
| S99 | TI crossover* OR AB crossover* |
| S100 | TI factorial* OR AB factorial* |
| S101 | TI random* OR AB random* |
| S102 | S91 OR S92 OR S93 OR S94 OR S95 OR S96 OR S97 OR S98 OR S99 OR S100 OR S101 |
| S103 | S61 AND S90 AND S102 |
| S104 | TI before N1 after OR AB before N1 after |
| S105 | TI pre N1 post OR AB pre N1 post |
| S106 | TI pretest N1 posttest OR AB pretest N1 posttest |
| S107 | TI "pre test" N1 "post test" OR AB "pre test" N1 "post test" |
| S108 | TI control* OR AB control* |
| S109 | DE "Time Series" |
| S110 | DE "Treatment Effectiveness Evaluation" |
| S111 | DE "Cohort Analysis" |
| S112 | TI cohort OR AB cohort |
| S113 | S104 OR S105 OR S106 OR S107 OR S108 OR S109 OR S110 OR S111 OR S112 |
| S114 | S9 OR S10 OR S11 OR S12 OR S13 OR S14 OR S15 OR S16 OR S17 OR S18 OR S19 OR S20 OR S21 OR S22 OR S23 OR S24 OR S25 OR S26 OR S27 |
| S115 | S90 AND S113 AND S114 |
| S116 | S103 OR S115 |

**Cochrane Central <1946 to October 1, 2024>**

| #1 | MeSH descriptor: [Chronic Disease] explode all trees |
| --- | --- |
| #2 | (chronic* NEAR/3 (condition* or disease* or disorder* or illness*)):ti,ab,kw |
| #3 | ("long term" NEAR/3 (condition* or disease* or disorder* or illness*)):ti,ab,kw |
| #4 | (longterm NEAR/3 (condition* or disease* or disorder* or illness*)):ti,ab,kw |
| #5 | ("long standing" NEAR/3 (condition* or disease* or disorder* or illness*)):ti,ab,kw |
| #6 | (longstanding NEAR/3 (condition* or disease* or disorder* or illness*)):ti,ab,kw |
| #7 | LTC*:ti,ab,kw |
| #8 | MeSH descriptor: [Rheumatic Diseases] this term only |
| #9 | MeSH descriptor: [Arthritis, Rheumatoid] explode all trees |
| #10 | MeSH descriptor: [Gout] explode all trees |
| #11 | MeSH descriptor: [Polymyalgia Rheumatica] this term only |
| #12 | MeSH descriptor: [Lupus Erythematosus, Systemic] this term only |
| #13 | MeSH descriptor: [Spondylarthropathies] explode all trees |
| #14 | MeSH descriptor: [Giant Cell Arteritis] this term only |
| #15 | (rheumat* NEAR/3 (arthrit* or disease* or condition* or nodule*)):ti,ab,kw |
| #16 | (inflamm* NEAR/3 (arthrit* or rheumat*)):ti,ab,kw |
| #17 | "ankylosing spondylitis":ti,ab,kw |
| #18 | "psoriatic arthritis":ti,ab,kw |
| #19 | polymyalgia:ti,ab,kw |
| #20 | arthropath*:ti,ab,kw |
| #21 | arthrosis:ti,ab,kw |
| #22 | arthroses:ti,ab,kw |
| #23 | Gout*:ti,ab,kw |
| #24 | polyarthropath*:ti,ab,kw |
| #25 | polyarthriti*:ti,ab,kw |
| #26 | PMR*:ti,ab,kw |
| #27 | pPsA:ti,ab,kw |
| #28 | axPsA:ti,ab,kw |
| #29 | "systemic lupus erythematosus":ti,ab,kw |
| #30 | axial spondy*:ti,ab,kw |
| #31 | AxSpA:ti,ab,kw |
| #32 | GCA*:ti,ab,kw |
| #33 | MeSH descriptor: [Osteoporosis] explode all trees |
| #34 | osteoporo*:ti,ab,kw |
| #35 | MeSH descriptor: [Diabetes Mellitus] explode all trees |
| #36 | diabet*:ti,ab,kw |
| #37 | MeSH descriptor: [Coronary Disease] explode all trees |
| #38 | (coronary NEAR/3 (condition* or disease* or disorder* or illness* or event*)):ti,ab,kw |
| #39 | MeSH descriptor: [Heart Diseases] explode all trees |
| #40 | (cardiac* NEAR/3 (condition* or disease* or disorder* or illness* or event* or arrest or arrhythmia*)):ti,ab,kw |
| #41 | "atrial fibrillation":ti,ab,kw |
| #42 | MeSH descriptor: [Heart Failure] explode all trees |
| #43 | (heart NEAR/3 (attack* or condition* or disease* or disorder* or fail* or insuff*)):ti,ab,kw |
| #44 | (myocard* NEAR/3 infarction):ti,ab,kw |
| #45 | MeSH descriptor: [Peripheral Arterial Disease] explode all trees |
| #46 | "peripheral arterial disease":ti,ab,kw |
| #47 | MeSH descriptor: [Stroke] explode all trees |
| #48 | Stroke*:ti,ab,kw |
| #49 | ((brain or cereb*) NEAR/3 (ish?emia or infarct*)):ti,ab,kw |
| #50 | (cerebrovascular NEAR/3 accident):ti,ab,kw |
| #51 | (acute NEAR/3 cerebrovascular):ti,ab,kw |
| #52 | CVA*:ti,ab,kw |
| #53 | "cerebral infarct*":ti,ab,kw |
| #54 | MeSH descriptor: [Ischemic Attack, Transient] this term only |
| #55 | TIA*:ti,ab,kw |
| #56 | "Transient ischemic":ti,ab,kw |
| #57 | MeSH descriptor: [Renal Insufficiency, Chronic] explode all trees |
| #58 | (kidney NEAR/3 (insufficien* or fail*)):ti,ab,kw |
| #59 | (renal NEAR/3 (insufficien* or fail*)):ti,ab,kw |
| #60 | (Chronic NEAR/3 (kidney or renal)):ti,ab,kw |
| #61 | CKD*:ti,ab,kw |
| #62 | MeSH descriptor: [Pulmonary Disease, Chronic Obstructive] explode all trees |
| #63 | emphysema*:ti,ab,kw |
| #64 | (chronic* NEAR/3 bronchiti*):ti,ab,kw |
| #65 | COPD:ti,ab,kw |
| #66 | COAD:ti,ab,kw |
| #67 | MeSH descriptor: [Asthma] explode all trees |
| #68 | asthma*:ti,ab,kw |
| #69 | MeSH descriptor: [Epilepsy] explode all trees |
| #70 | MeSH descriptor: [Seizures] explode all trees |
| #71 | (epilep*or seizure* or convuls*):ti,ab,kw |
| #72 | {OR #1-#71} |
| #73 | MeSH descriptor: [Depression] this term only |
| #74 | MeSH descriptor: [Depressive Disorder] explode all trees |
| #75 | MeSH descriptor: [Mood Disorders] this term only |
| #76 | MeSH descriptor: [Dysthymic Disorder] this term only |
| #77 | MeSH descriptor: [Anxiety] this term only |
| #78 | MeSH descriptor: [Anxiety Disorders] explode all trees |
| #79 | {OR #73-#78} |
| #80 | prevent*:ti,ab,kw |
| #81 | #79 AND #80 |
| #82 | MeSH descriptor: [Preventive Health Services] this term only |
| #83 | MeSH descriptor: [Primary Prevention] this term only |
| #84 | MeSH descriptor: [Secondary Prevention] this term only |
| #85 | {OR #82-#84} |
| #86 | (anxi* or depress*):ti,ab,kw |
| #87 | #79 OR #86 |
| #88 | #85 AND #87 |
| #89 | #81 OR #88 |
| #90 | (depress* NEAR/3 (reduc* or first episode* or onset* or prevent* or risk or at-risk or symptom*)):ti,ab,kw |
| #91 | (dysthymi* NEAR/3 (reduc* or first episode* or onset* or prevent* or risk or at-risk or symptom*)):ti,ab,kw |
| #92 | (affective disorder* NEAR/3 (reduc* or first episode* or onset* or prevent* or risk or at-risk or symptom*)):ti,ab,kw |
| #93 | (melanchol* NEAR/3 (reduc* or first episode* or onset* or prevent* or risk or at-risk or symptom*)):ti,ab,kw |
| #94 | (mood NEAR/3 (reduc* or first episode* or onset* or prevent* or risk or at-risk or symptom*)):ti,ab,kw |
| #95 | (depress* NEAR/3 (subclinical* or sub-clinical* or subthreshold* or sub-threshold* or subsyndrom* or sub-syndrom*)):ti,ab,kw |
| #96 | ((Mild* or minor) NEAR/3 depress*):ti,ab,kw |
| #97 | "low mood":ti,ab,kw |
| #98 | MeSH descriptor: [Quality of Life] this term only |
| #99 | MeSH descriptor: [Adaptation, Psychological] this term only |
| #100 | MeSH descriptor: [Psychosocial Intervention] this term only |
| #101 | {OR #98-#100} |
| #102 | depress*:ti |
| #103 | #102 AND #101 |
| #104 | (anxi* NEAR/3 (reduc* or first episode* or onset* or prevent* or risk or at-risk or symptom*)):ti,ab,kw |
| #105 | (panic* NEAR/3 (reduc* or first episode* or onset* or prevent* or risk or at-risk or symptom*)):ti,ab,kw |
| #106 | (stress* NEAR/3 (reduc* or first episode* or onset* or prevent* or risk or at-risk or symptom*)):ti,ab,kw |
| #107 | anxi*:ti |
| #108 | #107 AND #101 |
| #109 | (Anxi* OR depress*): ti AND prevent*:ti,ab,kw |
| #110 | #89 or #90 or #91 or #92 or #93 or #94 or #95 or #96 or #97 or #103 or #104 or #105 or #106 #108 or #109 |
| #111 | #72 AND #89 AND #110 |

**Web of Science <1946 to October 1, 2024>**

| 1 | ALL=(chronic disease) |
| --- | --- |
| 2 | TS=(longterm NEAR/3 (condition* or disease* or disorder* or illness*)) |
| 3 | TS=(chronic NEAR/3 (condition* or disease* or disorder* or illness*)) |
| 4 | TS=(longstanding NEAR/3 (condition* or disease* or disorder* or illness*)) |
| 5 | TS=LTC* |
| 6 | TS=(rheumat* NEAR/3 (arthrit* or disease* or condition* or nodule*)) |
| 7 | TS=(inflamm* NEAR/3 (arthrit* or rheumat*)) |
| 8 | TS=(“ankylosing spondylitis”) |
| 9 | TS=(“psoriatic arthritis”) |
| 10 | TS=(polymyalgia) |
| 11 | TS=(arthropath*) |
| 12 | TS=(arthrosis) |
| 13 | TS=(arthroses) |
| 14 | TS=(Gout*) |
| 15 | TS=(polyarthropath*) |
| 16 | TS=(polyarthritis*) |
| 17 | TS=(PMR*) |
| 18 | TS=(pPsA) |
| 19 | TS=(axPsA) |
| 20 | TS=(“systemic lupus erythematosus”) |
| 21 | TS=(“axial spondy*”) |
| 22 | TS=(AxSpA) |
| 23 | TS=(GCA*) |
| 24 | TS=(osteoporo*) |
| 25 | TS=(diabet*) |
| 26 | TS=(coronary NEAR/3 (condition* or disease* or disorder* or illness* or event*)) |
| 27 | TS=(“atrial fribrillation”) |
| 28 | TS=(heart NEAR/3 (attack* or condition* or disease* or disorder* or fail* or insuff*)) |
| 29 | TS=(myocard* NEAR/3 infarction) |
| 30 | TS=(“peripheral arterial disease”) |
| 31 | TS=(stroke) |
| 32 | TS=((brain or cereb*) NEAR/3 (ish?emia or infarct*)) |
| 33 | TS=(cerebrovascular NEAR/3 accident)) |
| 34 | TS=(acure NEAR/3 cerebrovascular) |
| 35 | TS=(CVA*) |
| 36 | TS=(cerebral infarct*) |
| 37 | TS=(TIA*) |
| 38 | TS=(“Transient ischemic”) |
| 39 | TS=(kidney NEAR/3 (insufficient* or fail*)) |
| 40 | TS=(renal NEAR/3 (insuficien* or fail*)) |
| 41 | TS=(chronic NEAR/3 (kidney or renal)) |
| 42 | TS=(CKD*) |
| 43 | TS=(emphysema*) |
| 44 | TS=(chronic* NEAR/3 bronchiti*) |
| 45 | TS=(COPD) |
| 46 | TS=(COAD) |
| 47 | TS=(asthma*) |
| 48 | TS=(epilep* or seizure* or convuls*) |
| 49 | **#48 OR #47 OR #46 OR #45 OR #44 OR #43 OR #42 OR #41 OR #40 OR #39 OR #38 OR #37 OR #36 OR #35 OR #34 OR #33 OR #32 OR #31 OR #30 OR #29 OR #28 OR #27 OR #26 OR #25 OR #24 OR #23 OR #22 OR #21 OR #20 OR #19 OR #18 OR #17 OR #16 OR #15 OR #14 OR #13 OR #12 OR #11 OR #10 OR #9 OR #8 OR #7 OR #6 OR #5 OR #4 OR #3 OR #2 OR #1** |
| 50 | **TS=(prevent*)** |
| 51 | **TS(anxi* or depress*)** |
| 52 | **TS=(depress* NEAR/3 (reduc* or “first episode”* or onset* or prevent* or risk* or at-risk or symptom*))** |
| 53 | **TS=(dysthymi* NEAR/3 (reduc* or “first episode”* or onset* or prevent* or risk* or at-risk or symptom*))** |
| 54 | **TS=(affective disorder* NEAR/3 (reduc* or “first episode”* or onset* or prevent* or risk* or at-risk or symptom*))** |
| 55 | **TS=(melancholy* NEAR/3 (reduc* or “first episode”* or onset* or prevent* or risk* or at-risk or symptom*))** |
| 56 | **TS=(mood NEAR/3 (reduc* or “first episode”* or onset* or prevent* or risk* or at-risk or symptom*))** |
| 57 | **TS=(depress* NEAR/3 (subclinical* or sub-clinical* or subthreshold* or sub-threshold* or subsyndrom* or sub-syndrom*))** |
| 58 | **TS=((Mild* or minor) NEAR/3 depress*)** |
| 59 | **TS=(“low mood”)** |
| 60 | **TS=(anxi* NEAR/3 (reduc* or “first episode”* or onset* or prevent* or risk* or at-risk or symptom*))** |
| 61 | **TS=(panic* NEAR/3 (reduc* or “first episode”* or onset* or prevent* or risk* or at-risk or symptom*))** |
| 62 | **TS=(stress* NEAR/3 (reduc* or “first episode”* or onset* or prevent* or risk* or at-risk or symptom*))** |
| 63 | **TS=(anxi* NEAR/3 (subclinical* or sub-clinical* or subthreshold* or sub-threshold* or subsyndrom* or sub-syndrom*))** |
| 64 | **#50 AND #51** |
| 65 | **#52 OR #53 OR #54 OR #55 OR #56 OR #57 OR #58 OR #59 OR #60 OR #61 OR #62 OR #63** |
| 66 | **#49 AND #65 AND #66** |
