# Supplementary material for: Impact of interventions to prevent anxiety and depression in people with inflammatory rheumatological conditions: a systematic review
Source: Rheumatol Adv Pract. 2026 May 29;10(3):rkag059. doi: 10.1093/rap/rkag059 (PMC13268797; doi:10.1093/rap/rkag059)
Supplement: rkag059_Supplementary_Data [file rkag059_supplementary_data.zip › Supplementary_Table_3_Intervention_Components_description.docx]

| Supplementary Table S3: Intervention Components and their descriptions | |
| --- | --- |
| Intervention Characteristics | Description |
| Problem Solving | This technique requires focus on real life scenarios and guiding participants to pursue the best course of action. Emphasis was place on identifying and dealing with the daily challenges of living with a condition |
| Cognitive Restructuring | This includes techniques that asks participants to reexamine their thoughts on living with their condition, identify the negative thoughts and work to challenge such thoughts to change irrational cognition and practice counterconditioning. |
| Attention Training | This enabled participants to lower their internal focus of attention, strengthen their external focus of attentions and thereby reduce worrying and rumination. |
| Communication Development | Includes building on communications with family, friends, romantic relationships and healthcare professionals. Communication skills building focuses on appropriate ways of expressing emotions respectfully to solicit support, as well as strategies to ask for assistance. |
| Mindfulness and Relaxation | Relaxation techniques such as deep breathing, progressive muscle relaxation and mindfulness practice were usually practiced as daily homework activities. |
| Exercise | Physical activities are used to improve mobility, pain and fitness including endurance training, aerobic exercise and yoga. |
| Reflection | Reflection helps participants balance a long-term condition alongside the rest of their lives. This included reflective practices in groups, reflective listening and reviewing wellbeing and mood diaries. |
| Peer Support | This involved people with the same lived experience sharing journeys and knowledge. |
| Education and Information | Includes education and informational resources which provided information on conditions including symptomology, medication and treatment options. But further self-management of symptoms, flare-ups, lifestyle adjustments and prevention of complications were also covered. |
| Homework | Assigning participants with tasks to be completed in between sessions. Some of these home activities were intended to reinforce any adaptive skills taught and encourage participant reflection (e.g., keeping a diary). |
| Finding Acceptance | The use of this helped participants accept the condition-related limitations imposed on them, as well as the instillation of hope and sense of purpose. Two interventions used ACT to achieve this. |
| Goal Setting | Goal settings involved making a list of tasks to be achieved and discussing with peers for encouragement and progress tracking. |
