# Supplementary material for: Impact of interventions to prevent anxiety and depression in people with inflammatory rheumatological conditions: a systematic review
Source: Rheumatol Adv Pract. 2026 May 29;10(3):rkag059. doi: 10.1093/rap/rkag059 (PMC13268797; doi:10.1093/rap/rkag059)
Supplement: rkag059_Supplementary_Data [file rkag059_supplementary_data.zip › Supplementary_Figure_1_Cochrane_Risk_of_Bias.docx]

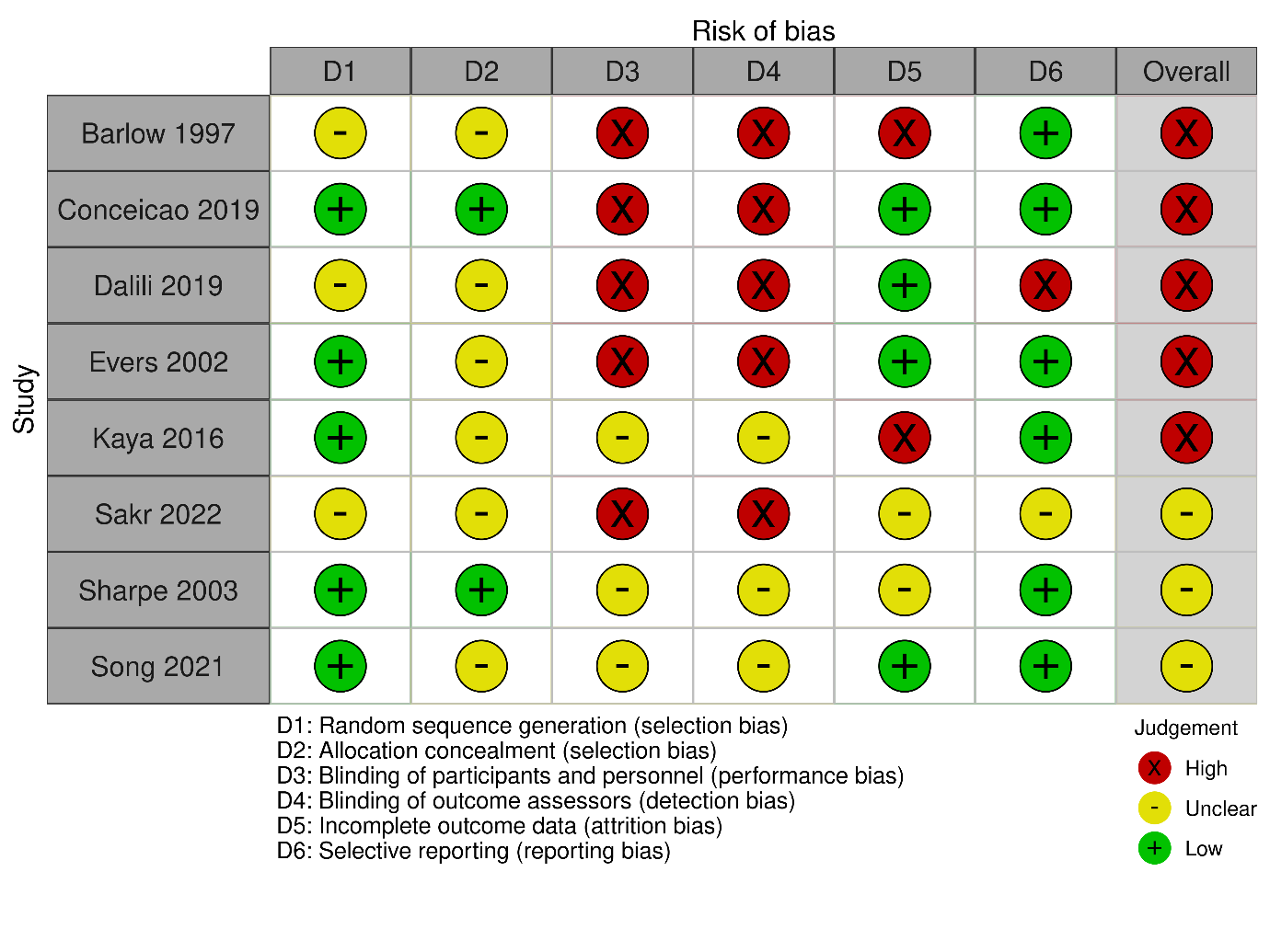
Supplementary Figure S1: Cochrane Risk of Bias

**Alt Text:** Graphical representation showing Risk of Bias for each study and individual domains, as well as overall Risk of Bias for each study.
